# Wavelength conversion through plasmon-coupled surface states

**DOI:** 10.1038/s41467-021-24957-1

**Published:** 2021-07-30

**Authors:** Deniz Turan, Ping Keng Lu, Nezih T. Yardimci, Zhaoyu Liu, Liang Luo, Joong-Mok Park, Uttam Nandi, Jigang Wang, Sascha Preu, Mona Jarrahi

**Affiliations:** 1 Electrical and Computer Engineering Department, University of California, Los Angeles, CA USA; 2 Department of Physics and Astronomy and Ames Laboratory-U.S. DOE, Iowa State University, Ames, IA USA; 3 Department of Electrical Engineering and Information Technology, Technical University Darmstadt, Darmstadt, Germany

**Keywords:** Nanoscale devices, Integrated optics, Optical materials and structures, Nanophotonics and plasmonics, Electronics, photonics and device physics

## Abstract

Surface states generally degrade semiconductor device performance by raising the charge injection barrier height, introducing localized trap states, inducing surface leakage current, and altering the electric potential. We show that the giant built-in electric field created by the surface states can be harnessed to enable passive wavelength conversion without utilizing any nonlinear optical phenomena. Photo-excited surface plasmons are coupled to the surface states to generate an electron gas, which is routed to a nanoantenna array through the giant electric field created by the surface states. The induced current on the nanoantennas, which contains mixing product of different optical frequency components, generates radiation at the beat frequencies of the incident photons. We utilize the functionalities of plasmon-coupled surface states to demonstrate passive wavelength conversion of nanojoule optical pulses at a 1550 nm center wavelength to terahertz regime with efficiencies that exceed nonlinear optical methods by 4-orders of magnitude.

## Abstract (web summary)

Semiconductor surface states often stand in the way of device performance, but here, the authors take advantage of them for wavelength conversion. They present a compact, passive conversion device insensitive to optical alignment by using plasmon-coupled surface states that enable the efficient conversion without nonlinear phenomena.

## Introduction

When a semiconductor lattice is terminated on the surface, the periodicity of the lattice is broken since the surface atoms do not have sufficient number of atoms that they can bond to, leaving behind incomplete chemical bonds. These so-called dangling bonds produce localized surface states with energy levels that are located within the bandgap of the semiconductor^1–4^. The Fermi energy level at the surface of a semiconductor is pinned to the energy level at which the surface state density peaks, while the Fermi energy level away from the semiconductor surface is determined by the semiconductor doping. Therefore, the presence of the surface states takes away a very important degree of freedom for engineering semiconductor devices by altering the electric potential profile and is generally a major source of degradation in semiconductor devices.

**Fig. 1:**
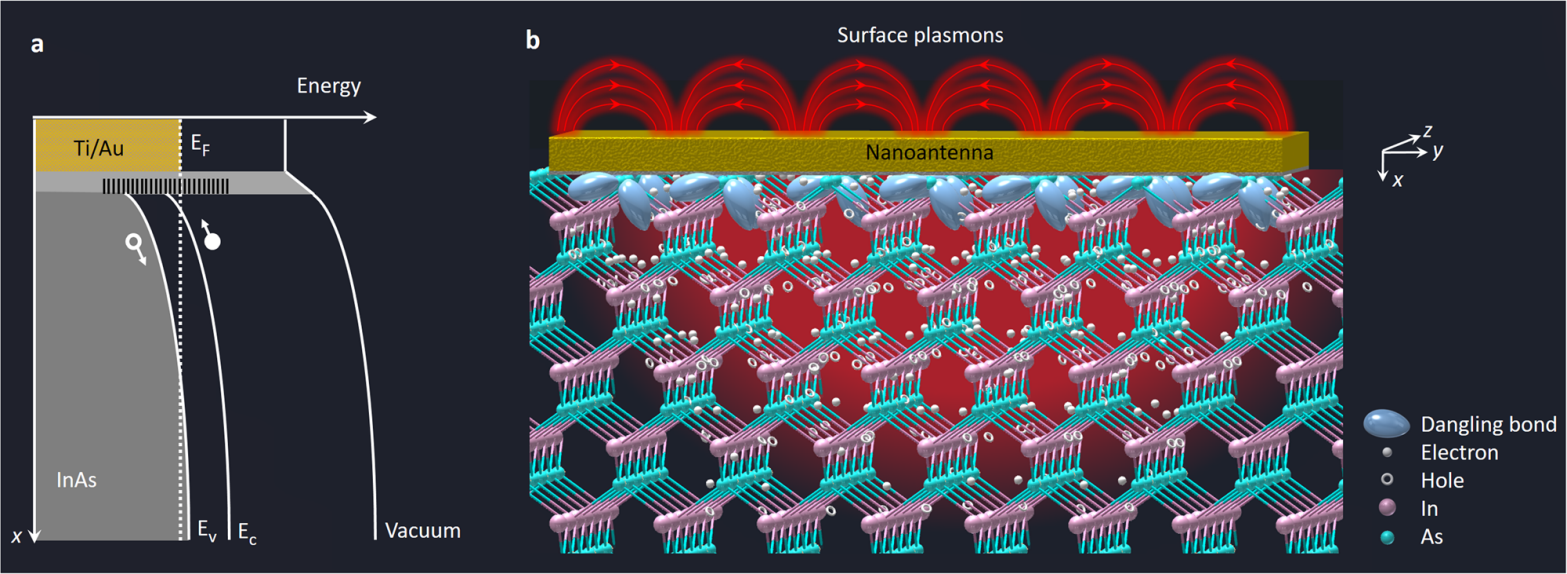
Energy band bending caused by the InAs surface states. **a** Energy band diagram of a highly p-doped InAs substrate in contact with Ti/Au. The energy level at which the surface state density of the InAs peaks is located above its direct bandgap because there is a large difference between the direct (0.36 eV) and indirect bandgap energies (1.21 eV) of InAs. Electrons in these surface states recombine with the holes in the valence band and occupy a part of the conduction band to minimize their total energy. As a result, the Fermi energy level (*E*_F_) is pinned above the conduction band minimum (*E*_*c*_). Free electrons in the conduction band then migrate to the p-doped InAs layer to minimize their energy further, resulting in a steep band bending and a giant built-in electric field induced at the InAs surface. **b** Schematic of the InAs lattice in contact with a nanoantenna that couples photo-excited surface plasmons to the surface states.

Despite the endless efforts to suppress the surface semiconductor states^5–9^, they have unique electrochemical properties that are not provided by bulk semiconductors and could enable unprecedented device functionalities. Figure 1a illustrates how the presence of surface states induces a giant built-in electric field at the surface of a p-doped InAs semiconductor, which exceeds the breakdown field of bulk InAs. The energy level at which the surface state density of InAs peaks is located above its direct bandgap because there is a large difference between the direct and indirect bandgap energies of InAs^2,3^. Since the electrons that occupy the surface states have an average total energy higher than the bulk InAs, they migrate from the surface states to the bulk InAs to reach equilibrium, leaving behind the immobile charge of uncompensated donor ions, which produces a giant built-in electric field. Ideally, wavelength conversion can be achieved by accelerating photoabsorbed charges through this giant built-in electric field with a very high mobility. However, efficient wavelength conversion was not possible before due to the very shallow band bending at the surface of the semiconductor, which severely limits the interaction between the giant built-in electric field and the optical beam. Instead, photo-Dember effect and nonlinear optical processes were the dominant mechanisms for passive wavelength conversion^10–13^.

In this work, we harness this shallow built-in electric field in InAs to perform optical wavelength conversion. To effectively utilize this built-in electric field, optical photons excite a nanoantenna array to couple photo-excited surface plasmons to the surface states (Fig. 1b). Excitation of surface plasmons enhances the optical intensity and photoabsorption near the InAs surface^14–26^, where the strength of the built-in electric field is maximized. The absorbed photons generate a tightly confined electron gas under the nanoantenna contacts with an electron concentration that resonates at the mixing product of different optical frequency components. This electron gas swiftly drifts to the nanoantennas through the built-in electric field. The induced current on the nanoantennas generates radiation at the beat frequencies of the optical photons.

## Results

### Passive optical-to-terahertz conversion

**Fig. 2:**
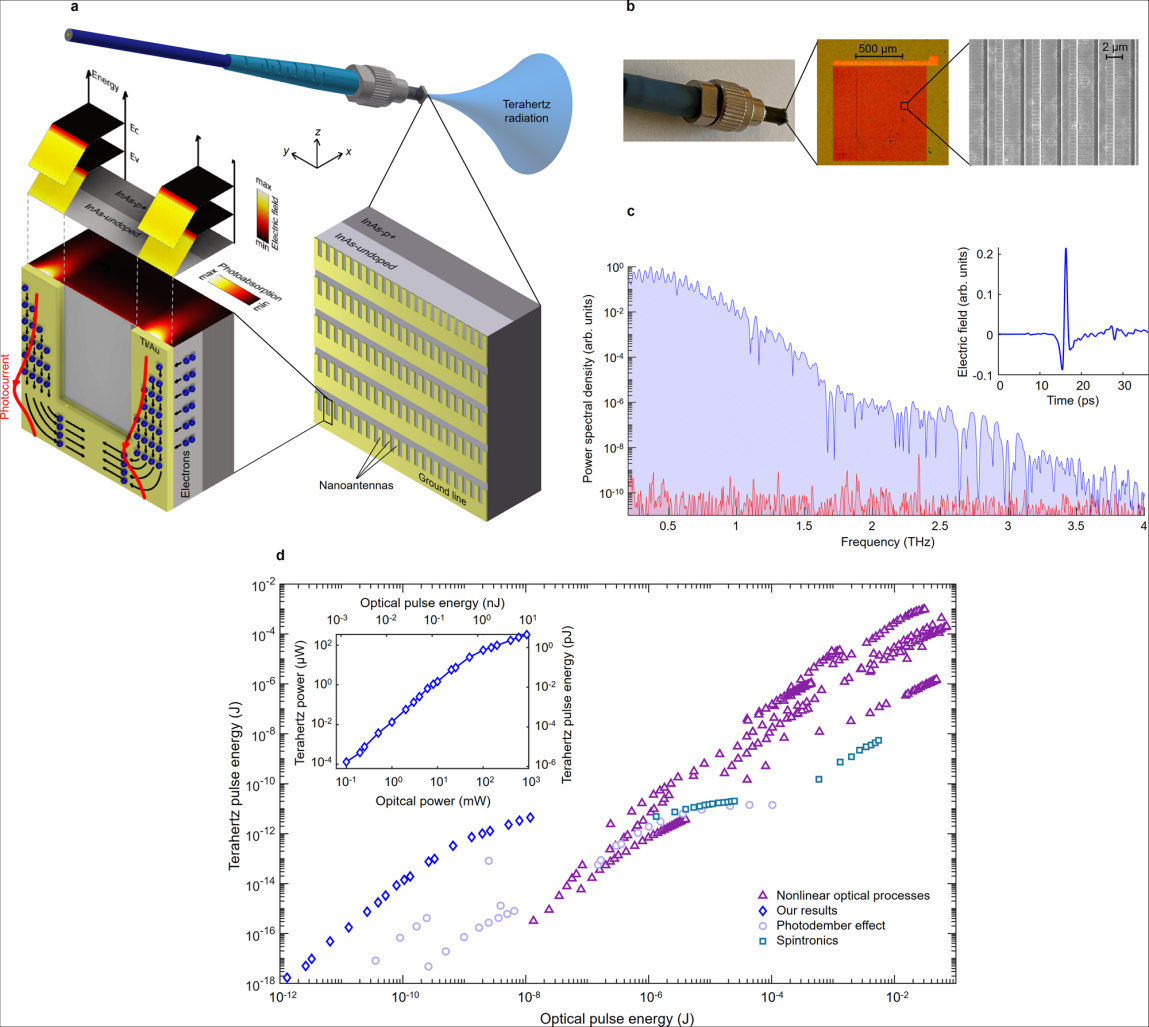
Wavelength conversion through plasmon-coupled surface states. **a** Schematic of a nanoantenna array on an InAs semiconductor substrate, which is designed to couple photo-excited surface plasmons to the surface states where a built-in electric field drifts the photo-induced electron gas to the nanoantennas to generate radiation at the optical beat frequencies. The nanoantenna geometry and semiconductor structure are chosen to maximize the spatial overlap between the built-in electric field and photoabsorption profiles. Three-dimensional computer aided design (CAD) drawing of the fiber connector (item no: 30125D2) provided as compliments by Thorlabs, Inc. **b** Photograph, microscopy, and scanning electron microscopy images of a fabricated nanoantenna array on a substrate consisting of a 100-nm-thick undoped InAs layer grown on a 500-nm-thick InAs epilayer with a p-type doping of 10^19^ cm^−3^ grown on a semi-insulating GaAs substrate. **c** Measured terahertz radiation (in blue) and noise (in red) spectra generated from the nanoantenna array when pumped by 3.68 nJ optical pulses at a 1550 nm center wavelength. The time-domain radiated terahertz pulse is shown in the inset. A total of 3200 time-domain traces are captured and averaged to resolve this spectrum. **d** Measured terahertz pulse energy/power from the fabricated nanoantenna array as a function of the optical pulse energy/power (inset) in comparison with other passive optical-to-terahertz converters reported in the literature. The optical pulse energy/power range of the available laser for these measurements was ~10 nJ/1 W. A linear dependence between the terahertz and optical pulse energy/power levels is expected at higher pulse energy/power levels (Supplementary Fig. 4). A comparison with active optical-to-terahertz converters reported in the literature is provided in Supplementary Fig. 5.

Figure 2a shows a nanoantenna array designed to couple photo-excited surface plasmons to the InAs surface states where a built-in electric field drifts the photo-induced electron gas to the nanoantennas to generate radiation at the optical beat frequencies. Unlike the bulky and complex nonlinear optical setups that require high-energy lasers, tight optical focus, and/or tilted beam to provide high optical pump intensity and phase matching for efficient wavelength conversion, wavelength conversion through plasmon-coupled surface states does not require a complex optical setup and is not sensitive to optical focus and alignment (Supplementary Fig. 1). Figure 2b shows a fabricated nanoantenna array on InAs that is simply glued at the tip of an optical fiber without using any intermediate optical component and can be pumped by a compact fiber laser.

We experimentally demonstrate the conversion of 3.68 nJ optical pulses with a 150 fs pulsewidth coupled to the fiber at a 1550 nm center wavelength to 1.78 pJ terahertz pulses radiated from the nanoantenna array (Supplementary Fig. 2) with more than a 4 THz bandwidth and 105 dB dynamic range (Fig. 2c). Broader radiation bandwidths exceeding 6 THz and higher dynamic ranges exceeding 110 dB are achieved when using the optical pulses with shorter pulsewidth and higher power (Supplementary Fig. 3). The measured terahertz pulse energy/power from the fabricated nanoantenna array as a function of the optical pulse energy/power (Fig. 2d inset) is compared with other passive optical-to-terahertz converters reported in the literature, which utilize nonlinear optical processes^27–47^, spintronics^48–51^, and the photo-Dember effect^52–55^. The comparison indicates a record-high efficiency of the plasmon-coupled surface states in passive wavelength conversion of nanojoule optical pulses to terahertz regime with efficiencies that exceed nonlinear optical methods by the 4 orders of magnitude (Fig. 2d).

### Engineering the semiconductor band bending

**Fig. 3:**
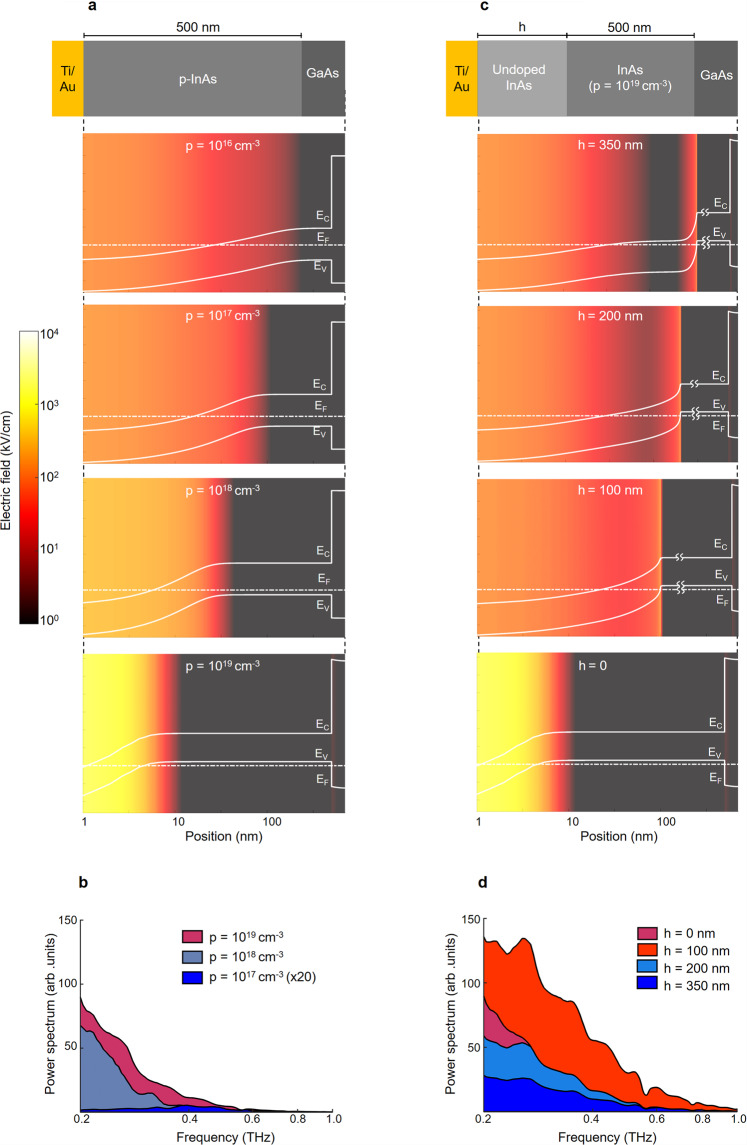
Built-in electric field profile and its impact on the wavelength conversion efficiency. **a** Band diagram of the p-doped InAs layer below the Ti/Au nanoantenna contact at different p-type doping concentrations are shown in white. The color map shows the strength of the built-in electric field. A Sentaurus device simulator is used to generate the band diagram and built-in electric field plots. The built-in electric field drifts the high-mobility photo-generated electrons to the Ti/Au contact without any barrier height and sweeps away the low-mobility photo-generated holes from the Ti/Au contact. **b** The measured terahertz radiation spectra from identical nanoantenna arrays fabricated on three InAs substrates with the p-type doping concentrations of 10^17^, 10^18^, and 10^19^ cm^−3^ in response to the same optical pump beam. The radiation spectra are shown in a linear scale to clearly show the wavelength conversion efficiency variations. **c** Band diagram and the built-in electric field profiles; when an undoped InAs layer is incorporated between the p-doped InAs epilayer and the Ti/Au contact. **d** The measured terahertz radiation spectra from identical nanoantenna arrays fabricated on four InAs substrates with undoped InAs layer thicknesses of 0, 100, 200, and 350 nm grown on an InAs epilayer with a p-type doping of 10^19^ cm^−3^.

In order to achieve high wavelength conversion efficiencies, the semiconductor structure and nanoantenna geometry are chosen to maximize the spatial overlap between the built-in electric field and photoabsorption profiles. The strength and extent of the built-in electric field below the InAs surface can be controlled by the doping profile of the InAs substrate. As illustrated in Fig. 3a, since the Fermi energy level at the surface of InAs is pinned above the conduction band minimum, increasing the p-type doping of the bulk results in a steeper band bending and, therefore, a stronger built-in electric field near the InAs surface. To better show the impact of the substrate doping, identical nanoantenna arrays are fabricated on three InAs substrates with p-type doping concentrations of 10^17^, 10^18^, and 10^19^ cm^−3^ and their optical-to-terahertz conversion performance is characterized under the same optical pump beam. As predicted by the energy band diagrams illustrated in Fig. 3a, the nanoantenna array fabricated on the InAs substrate with a p-type doping concentration of 10^19^ cm^−3^ offers the highest wavelength conversion efficiency among the three as it benefits from the highest built-in electric field near the InAs surface (Fig. 3b).

However, increasing the p-type doping reduces the extent of the built-in electric field below the InAs surface and lowers the spatial overlap between the built-in electric field and photoabsorption profiles. One way to extend the built-in electric field below the InAs surface is by incorporating an undoped InAs layer between the p-doped InAs epilayer and the nanoantenna contact. As illustrated in Fig. 3c, increasing the thickness of the undoped InAs layer further extends the band bending below the InAs surface while reducing the band bending slope, indicating a trade-off between the strength and extent of the built-in electric field in the substrate. To better show the impact of this trade-off, identical nanoantenna arrays are fabricated on four InAs substrates with undoped InAs layer thicknesses of 0, 100, 200, and 350 nm grown on an InAs epilayer with a p-type doping of 10^19^ cm^−3^ and their optical-to-terahertz conversion performance is characterized under the same optical pump beam. As demonstrated in Fig. 3d, the use of a 100-nm-thick undoped InAs layer increases the wavelength conversion efficiency by extending the built-in electric field in the semiconductor and increasing its spatial overlap with the photoabsorption profile. However, further increase in the thickness of the undoped InAs layer lowers the wavelength conversion efficiency due to the reduction in the built-in electric field strength. Because of its high wavelength conversion efficiency, the nanoantenna array fabricated on a 100-nm-thick undoped InAs layer grown on an InAs epilayer with a p-type doping of 10^19^ cm^−3^ is used to demonstrate the results shown in Fig. 2.

### Design of the plasmonic nanoantenna array

**Fig. 4:**
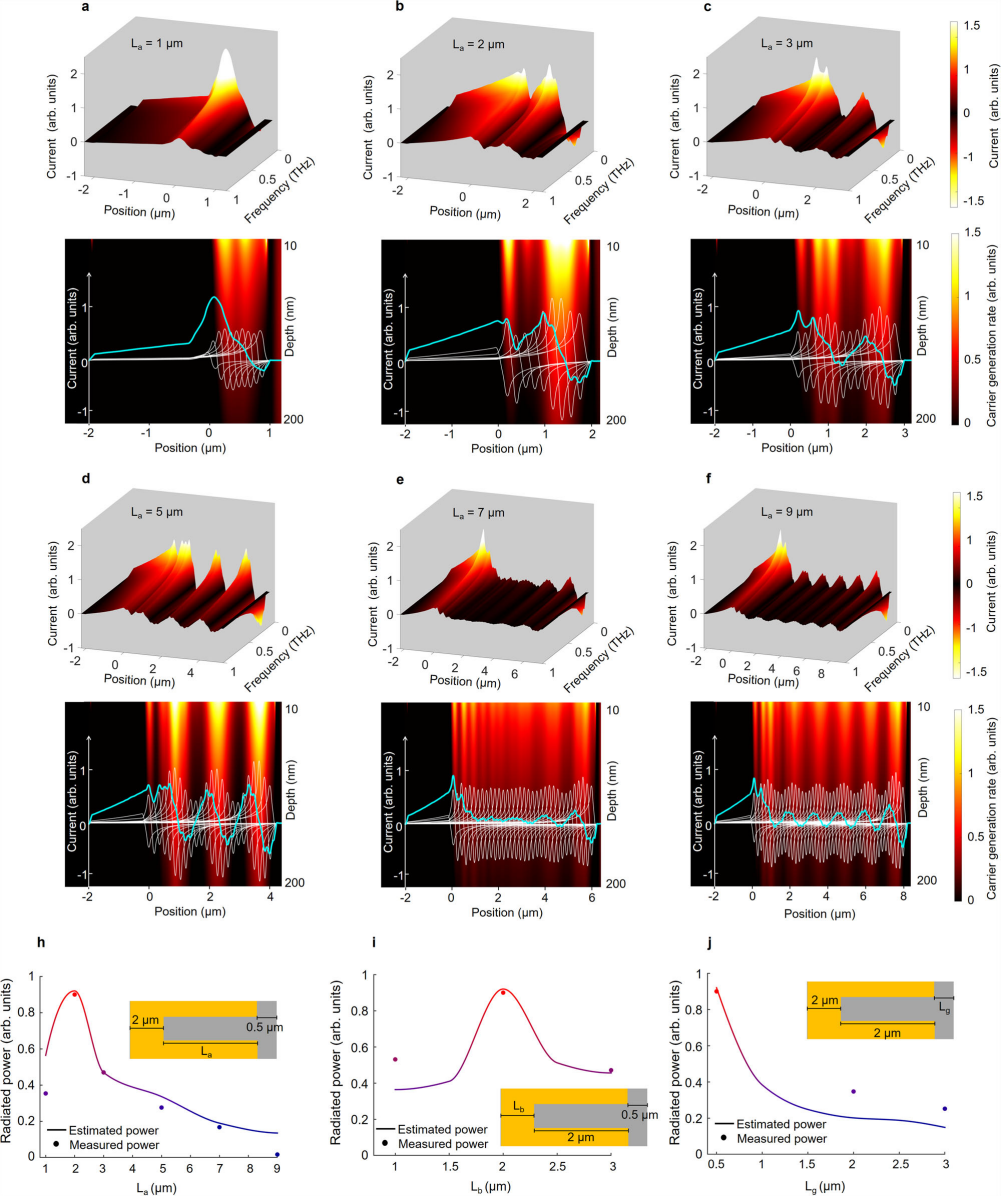
Impact of the nanoantenna geometry on the wavelength conversion efficiency. **a**–**f** Top: The induced current on the nanoantennas as a function of frequency when the nanoantenna length (*L*_a_) is varied from 1 to 9 μm. The ground line width (*L*_b_) and the gap between the nanoantenna array rows (*L*_g_) are chosen as 2 and 0.5 μm. The ground line is located between −2 μm and 0 positions and the nanoantenna is located between 0 and 1–9 μm positions along the z-axis. **a**–**f** Bottom: Decomposition of the total induced current on the nanoantennas (teal lines) to the individual contributions of the injected currents from different positions of the nanoantennas (white lines) at 0.2 THz. The background color maps show the electron generation profiles averaged over the nanoantenna width. Predicted and measured terahertz radiation power from fabricated nanoantenna arrays with different nanoantenna lengths, ground line widths, and gap sizes between the nanoantenna rows are shown in **h**, **i**, and **j** respectively. All the fabricated nanoantenna arrays have a 1 × 1 mm^2^ area and are characterized using the same optical pulses with 120 fs pulse width, 2.63 nJ pulse energy, and 76 MHz repetition rate.

The periodicity of the nanoantennas in the y-direction is chosen as 440 nm to provide the necessary momentum to couple the photo-excited surface plasmon waves to the interface between the metal contact and InAs substrate when excited by a TM-polarized optical beam at 1550 nm wavelength (Supplementary Fig. 6). A 240-nm-thick Si_3_N_4_ anti-reflection coating, a 360-nm-thick nanoantenna width, and a 3/97-nm-thick Ti/Au nanoantenna height are used to increase the coupling efficiency of surface plasmon waves. The geometry of the nanoantenna array is chosen to provide high-efficiency radiation over a broad terahertz frequency range when fed with the injected electrons from the InAs substrate. Radiation power is calculated from the induced current on the nanoantennas^56^. A finite-element-method-based electromagnetic solver (ANSYS-HFSS) is used to compute the induced current on nanoantennas for various geometrical parameters as a function of frequency. Figure 4a–f (top) shows the induced current on the nanoantennas for different nanoantenna lengths (*L*_a_) varying between 1 and 9 μm as a function of frequency. The steady reduction in the current amplitudes at higher frequencies is due to the non-zero transit time of the photogenerated electrons in InAs to the nanoantennas, which determines the photocurrent impulse response (Supplementary Fig. 8). Figure 4a–f (bottom) shows the decomposition of the total induced current on the nanoantennas (teal lines) to the individual contributions of the injected currents from different positions of the nanoantennas (white lines) at 0.2 THz. The background color maps show the electron generation profiles computed using a finite-difference time-domain electromagnetic solver (Lumerical), averaged over the nanoantenna width. As expected, the induced current at a different nanoantenna location is proportional to the electron generation rate, which causes the ripples observed in the total induced currents. The current that is injected near the nanoantenna tip and the nanoantenna-ground line intersection has the highest contribution to the total induced current on the nanoantennas. As the injection point is moved from these margins, the induced current splits into two near-equal current components that are 180° out-of-phase from one another, resulting in a destructive radiation from these out-of-phase current components (Supplementary Fig. 10). As the nanoantenna length is decreased from 9 (Fig. 4f) to 2 μm (Fig. 4b), the regions on the nanoantenna that do not contribute to the radiation are eliminated and the current density on the nanoantennas is increased, resulting in higher radiation powers. When the antenna length is reduced below 2 μm, the injected current to the nanoantenna is reduced because the ground lines shadow a major fraction of the optical beam, reducing the number of the photogenerated electrons in InAs (Fig. 4a). Apart from the nanoantenna length, the ground line width (*L*_b_) and the gap between the nanoantenna array rows (*L*_g_) also have a significant impact on the radiation efficiency (Supplementary Figs. 11 and 12). Increasing the width of the ground lines provides a lower impedance ground path for the current flow through the nanoantennas, resulting in an increase in the induced current. However, increasing the ground line width beyond 2 μm reduces the injected current to the nanoantenna because the ground lines shadow a major fraction of the optical beam, reducing the number of the photogenerated electrons in InA. Additionally, since the photogenerated electrons inside the gap between the nanoantenna array rows do not contribute to the radiation, this gap should be kept very small to maximize the fill factor of the radiating elements. To better show the impact of the nanoantenna geometry, nanoantenna arrays with different nanoantenna lengths, ground line widths, and gap sizes between the nanoantenna rows are fabricated with a total area of 1 × 1 mm^2^ and their radiation power is characterized under the same femtosecond optical pulse illumination. As illustrated in Fig. 4h–j, the measured terahertz radiation powers are in agreement with the theoretical predictions based on the induced current profiles on the nanoantennas.

## Discussion

Nonlinear optical materials have been the best known means for passive wavelength conversion, serving as the key enabler in many breakthrough technologies such as optical sources, processors, sensors, imaging, and quantum optical systems. This work introduces a fundamentally different paradigm for passive wavelength conversion with orders of magnitude higher efficiencies compared to nonlinear optical processes by utilizing the naturally induced built-in electric field in semiconductor surface states, which was not possible before due to the extremely shallow nature of these states. The significant enhancement in wavelength conversion efficiency combined with the physical attributes of plasmon-coupled surface states enable new functionalities that cannot be offered by nonlinear optical processes. Unlike nonlinear optical processes, wavelength conversion through plasmon-coupled surface states is not restricted by the Manley−Rowe limit, enabling access to new parts of the electromagnetic spectrum (e.g., millimeter-wave, microwave, and radio frequencies) that were not accessible before through nonlinear optical processes. In addition, plasmon-coupled surface states allow passive wavelength conversion through materials that do not support many of the nonlinear optical processes. For example, semiconductor lattices that possess axial symmetry (e.g. silicon and germanium) cannot provide second-order nonlinearity. However, wavelength conversion in these materials is possible by the use of plasmon-coupled surface states, opening up opportunities for new sensing, imaging, communication, and computation platforms compatible with standard integrated circuit technologies. The enhancement in wavelength conversion efficiency offered by the plasmon-coupled surface states significantly reduces the required optical power budget, enabling operation in compact and miniaturized system platforms that are not possible for the nonlinear optical systems. As an example, the demonstrated fiber-coupled nanoantenna chip sitting at a tip of a fiber (Fig. 2) can be easily integrated with an endoscopy probe for in vivo terahertz imaging and spectroscopy. To achieve the same terahertz power levels through nonlinear optical processes, 4-orders of magnitude higher optical power is required, which cannot be supported by optical fibers. Moreover, unlike the bulky and complex nonlinear optical setups that require high-energy lasers, tight optical focus, and/or tilted beam to provide high optical pump intensity and phase matching for efficient wavelength conversion, wavelength conversion through plasmon-coupled surface states does not require a complex optical setup and is not sensitive to optical focus and alignment, extending the scope of its potential use for many practical applications. Furthermore, plasmon-coupled surface states allow passive wavelength conversion using nanoantenna arrays, which can manipulate the spatial, spectral, and polarization state of the generated electromagnetic waves, which are not possible by other passive wavelength conversion techniques (nonlinear optical processes, spintronics, photo-Dember effect).

The presented wavelength conversion scheme based on plasmon-coupled surface states can be used for optical wavelength conversion to different parts of the electromagnetic spectrum ranging from microwave to far-infrared regimes in both pulsed and continuous wave operation (Supplementary Fig. 13). The wavelength conversion efficiency can be further enhanced by boosting the built-in electric field at the semiconductor surface and increasing the spatial overlap between the built-in electric field and photoabsorption profiles. Using alternative semiconductors with a larger number of surface states above the conduction band, introducing higher p-type doping levels, and incorporating a gradient composition semiconductor (In_1-x_Ga_x_As with x increasing as a function of depth in the substrate) would introduce a steeper band bending at the semiconductor surface and, therefore, would further enhance the built-in electric field. In addition, by growing the semiconductor active layer on a distributed Bragg reflector and further optimization of the nanoantenna geometry, most of the excited surface plasmons would be trapped in the semiconductor active layer and, therefore, a much stronger spatial overlap between the built-in electric field and photoabsorption profiles can be achieved (Supplementary Figs. 14 and 15).

## Methods

### Semiconductor growth

InAs layers are grown by molecular beam epitaxy (Veeco GEN-930) on semi-insulating GaAs (001) substrates. The growth is performed in an As-rich chamber at 400 °C. Be is used to dope the InAs to achieve a p-type doping concentration of 10^19^ cm^−3^.

### Device fabrication

The nanoantenna arrays are first defined using electron-beam lithography (Vistec EBPG 5000+ES) followed by 3/97 nm Ti/Au evaporation (CHA solution electron-beam evaporator) and lift-off. Ground lines are defined by electron-beam lithography followed by a 40/360 nm Ti/Au evaporation and lift-off. Finally, a 240-nm-thick Si_3_N_4_ anti-reflection coating is deposited using plasma-enhanced chemical vapor deposition (STS Multiplex CVD).

### Radiation measurements

An optical parametric oscillator (OPO) pumped by a Ti:sapphire laser (Coherent Mira-HP) is used to pump the fabricated nanoantenna arrays. It provides optical pulses at a 1550 nm central wavelength, 76 MHz repetition rate, and 120 fs pulsewidth. To demonstrate the fiber-coupled wavelength conversion performance shown in Fig. 2, the optical beam from the OPO is coupled to a polarization maintaining optical fiber (Thorlabs PM1550-XP) and the nanoantenna array is glued at the tip of the fiber. The optical pulsewidth incident on the nanoantenna array is increased to 150 fs due to the fiber dispersion. Terahertz radiation power measurements are performed using a calibrated pyroelectric detector (Sensors und Lasertechnik THz-30 detector calibrated by Physikalisch-Technische Bundesanstalt, Germany). Terahertz radiation spectrum measurements are performed using a terahertz time-domain spectroscopy setup with an ErAs:InGaAs-based photoconductive dipole antenna used as the terahertz detector^57^. The detector current is fed to a transimpedance amplifier (FEMTO DHPCA amplifier with an amplifier gain of 10^6^ V/A and a bandwidth of 1.8 MHz) followed by a lock-in amplifier (Zurich Instruments MFLI). Up to a 110 dB dynamic range and radiation up to 5 THz is achieved at a 900 mW optical power when using 1550 nm optical pulses with a 120 fs pulsewidth and a 76 MHz repetition rate (Supplementary Fig. 1). The terahertz radiation bandwidth can be further extended by using optical pulses with narrower pulsewidths. As shown in Supplementary Fig. 2, a terahertz radiation bandwidth exceeding 6.5 THz is measured when using 1560 nm optical pulses with a 23 fs pulsewidth, 100 mW average power, and a 40 MHz repetition rate (Er-doped fiber laser amplifier, TOPTICA Photonics FemtoFiber pro IRS-II).

## Supplementary information

Supplementary Information

Description of Additional Supplementary Files

Supplementary Video 1

Supplementary Video 2

## Data Availability

All the data and methods are present in the main text and the supplementary materials. Any other relevant data are available from the authors upon reasonable request.

## Code availability

Codes used in this work are available from the authors upon reasonable request.

## References

[1] Mönch W (1990). On the physics of metal-semiconductor interfaces. Rep. Prog. Phys..

[2] Tersoff J (1985). Schottky barriers and semiconductor band structures. Phys. Rev. B.

[3] Piper LFJ, Veal TD, Lowe MJ, McConville CF (2006). Electron depletion at InAs free surfaces: doping-induced acceptorlike gap states. Phys. Rev. B.

[4] Mönch, W. *Semiconductor Surfaces and Interfaces* (Springer-Verlag, 1993).

[5] Liu Z, Kobayashi M, Paul BC, Bao Z, Nishi Y (2010). Contact engineering for organic semiconductor devices via Fermi level depinning at the metal-organic interface. Phys. Rev. B..

[6] Suyatin DB (2014). Strong Schottky barrier reduction at Au-catalyst/GaAs-nanowire interfaces by electric dipole formation and Fermi-level unpinning. Nat. Commun..

[7] Liu Y, Stradins P, Wei S-H (2016). Van der Waals metal-semiconductor junction: Weak Fermi level pinning enables effective tuning of Schottky barrier. Sci. Adv..

[8] Chen YH (2019). Oxidized-monolayer tunneling barrier for strong Fermi-level depinning in layered InSe transistors. npj 2D Mater Appl.

[9] Savich GR, Pedrazzani JR, Maimon S, Wicks GW (2010). Suppression of surface leakage currents using molecular beam epitaxy-grown unipolar barriers. J. Vac. Sci. Technol. B.

[10] Adomavičius R, Urbanowicz A, Molis G, Krotkus A, Šatkovskis E (2004). Terahertz emission from p-lnAs due to the instantaneous polarization. Appl. Phys. Lett..

[11] Johnston MB, Whittaker DM, Corchia A, Davies AG, Linfield EH (2002). Simulation of terahertz generation at semiconductor surfaces. Phys. Rev. B.

[12] Liu K, Xu J, Yuan T, Zhang XC (2006). Terahertz radiation from InAs induced by carrier diffusion and drift. Phys. Rev. B.

[13] Tonouchi M (2020). Simplified formulas for the generation of terahertz waves from semiconductor surfaces excited with a femtosecond laser. J. Appl. Phys..

[14] Atwater HA, Polman A (2010). Plasmonics for improved photovoltaic devices. Nat. Mater..

[15] Schuller JA (2010). Plasmonics for extreme light concentration and manipulation. Nat. Mater..

[16] Cubukcu E, Kort EA, Crozier KB, Capasso F (2006). Plasmonic laser antenna. Appl. Phys. Lett..

[17] Wang N, Cakmakyapan S, Lin YJ, Javadi H, Jarrahi M (2019). Room-temperature heterodyne terahertz detection with quantum-level sensitivity. Nat. Astron..

[18] Berry CW, Wang N, Hashemi MR, Unlu M, Jarrahi M (2013). Significant performance enhancement in photoconductive terahertz optoelectronics by incorporating plasmonic contact electrodes. Nat. Commun..

[19] Brongersma ML, Halas NJ, Nordlander P (2015). Plasmon-induced hot carrier science and technology. Nat. Nanotechnol..

[20] Knight MW, Sobhani H, Nordlander P, Halas NJ (2011). Photodetection with active optical antennas. Science.

[21] Rastinehad AR (2019). Gold nanoshell-localized photothermal ablation of prostate tumors in a clinical pilot device study. Proc. Natl Acad. Sci. USA.

[22] Aslam U, Chavez S, Linic S (2017). Controlling energy flow in multimetallic nanostructures for plasmonic catalysis. Nat. Nanotechnol..

[23] Oulton RF (2009). Plasmon lasers at deep subwavelength scale. Nature.

[24] Noginov MA (2009). Demonstration of a spaser-based nanolaser. Nature.

[25] Lu YJ (2012). Plasmonic nanolaser using epitaxially grown silver film. Science.

[26] Pala RA, White J, Barnard E, Liu J, Brongersma ML (2009). Design of plasmonic thin-film solar cells with broadband absorption enhancements. Adv. Mater..

[27] Hauri CP, Ruchert C, Vicario C, Ardana F (2011). Strong-field single-cycle THz pulses generated in an organic crystal. Appl. Phys. Lett..

[28] Brunner FDJ (2008). A hydrogen-bonded organic nonlinear optical crystal for high-efficiency terahertz generation and detection. Opt. Express.

[29] Hoffmann MC, Yeh K-L, Hebling J, Nelson KA (2007). Efficient terahertz generation by optical rectification at 1035 nm. Opt. Express.

[30] Yeh KL, Hoffmann MC, Hebling J, Nelson KA (2007). Generation of 10 μJ ultrashort terahertz pulses by optical rectification. Appl. Phys. Lett..

[31] Blanchard F (2007). Generation of 1.5 µJ single-cycle terahertz pulses by optical rectification from a large aperture ZnTe crystal. Opt. Express.

[32] Chang G (2006). Power scalable compact THz system based on an ultrafast Yb-doped fiber amplifier. Opt. Express.

[33] Jewariya M, Nagai M, Tanaka K (2009). Enhancement of terahertz wave generation by cascaded χ2 processes in LiNbO3. J. Opt. Soc. Am. B.

[34] Stepanov AG, Bonacina L, Chekalin SV, Wolf J-P (2008). Generation of 30 μJ single-cycle terahertz pulses at 100 Hz repetition rate by optical rectification. Opt. Lett..

[35] Hirori H, Doi A, Blanchard F, Tanaka K (2011). Single-cycle terahertz pulses with amplitudes exceeding 1 MV/cm generated by optical rectification in LiNbO3. Appl. Phys. Lett..

[36] Fülöp JA (2012). Generation of sub-mJ terahertz pulses by optical rectification. Opt. Lett..

[37] Fülöp JA (2014). Efficient generation of THz pulses with 0.4 mJ energy. Opt. Express.

[38] Blanchard F (2014). Effect of extreme pump pulse reshaping on intense terahertz emission in lithium niobate at multimilliJoule pump energies. Opt. Lett..

[39] Ruchert C, Vicario C, Hauri CP (2013). Spatiotemporal focusing dynamics of intense supercontinuum THz pulses. Phys. Rev. Lett..

[40] Wu X (2018). Highly efficient generation of 0.2 mJ terahertz pulses in lithium niobate at room temperature with sub-50 fs chirped Ti:sapphire laser pulses. Opt. Express.

[41] Ruchert C, Vicario C, Hauri CP (2012). Scaling submillimeter single-cycle transients toward megavolts per centimeter field strength via optical rectification in the organic crystal OH1. Opt. Lett..

[42] Vicario C, Ovchinnikov AV, Ashitkov SI, Agranat MB, Hauri CP (2014). Generation of 0.9-mJ THz pulses in DSTMS pumped by a Cr:Mg_2_SiO_4_ laser. Opt. Lett..

[43] Rovere A (2018). Generation of high-field terahertz pulses in an HMQ-TMS organic crystal pumped by an ytterbium laser at 1030 nm. Opt. Express.

[44] Lu J (2018). Efficient terahertz generation in highly nonlinear organic crystal HMB-TMS. Opt. Express.

[45] Huang WR (2015). Highly efficient terahertz pulse generation by optical rectification in stoichiometric and cryo-cooled congruent lithium niobate. J. Mod. Opt..

[46] Stepanov AG, Hebling J, Kuhl J (2003). Efficient generation of subpicosecond terahertz radiation by phase-matched optical rectification using ultrashort laser pulses with tilted pulse fronts. Appl. Phys. Lett..

[47] Stepanov AG (2005). Scaling up the energy of THz pulses created by optical rectification. Opt. Express.

[48] Seifert T (2017). Ultrabroadband single-cycle terahertz pulses with peak fields of 300 kV cm-1 from a metallic spintronic emitter. Appl. Phys. Lett..

[49] Yang D (2016). Powerful and Tunable THz Emitters Based on the Fe/Pt Magnetic Heterostructure. Adv. Opt. Mater..

[50] Nandi U (2019). Antenna-coupled spintronic terahertz emitters driven by a 1550 nm femtosecond laser oscillator. Appl. Phys. Lett..

[51] Seifert T (2016). Efficient metallic spintronic emitters of ultrabroadband terahertz radiation. Nat. Photonics.

[52] Klatt G (2011). Photo-Dember terahertz emitter excited with an Er:fiber laser. Appl. Phys. Lett..

[53] Smith ML, Mendis R, Vickers REM, Lewis RA (2009). Comparison of photoexcited p-InAs THz radiation source with conventional thermal radiation sources. J. Appl. Phys..

[54] Reid M, Fedosejevs R (2005). Quantitative comparison of terahertz emission from (100) InAs surfaces and a GaAs large-aperture photoconductive switch at high fluences. Appl. Opt..

[55] Johnston MB (2002). Generation of high-power terahertz pulses in a prism. Opt. Lett..

[56] Balanis, C. *Antenna Theory; Analysis and Design* (John Wiley & Sons Inc, 2005).

[57] Nandi U, Norman JC, Gossard AC, Lu H, Preu S (2018). 1550-nm Driven ErAs:In(Al)GaAs photoconductor-based Terahertz time domain system with 6.5 THz bandwidth. J. Infrared, Millim. Terahertz Waves.

