# Author Correction: Wavelength conversion through plasmon-coupled surface states

**DOI:** 10.1038/s41467-021-26064-7

**Published:** 2021-09-29

**Authors:** Deniz Turan, Ping Keng Lu, Nezih T. Yardimci, Zhaoyu Liu, Liang Luo, Joong-Mok Park, Uttam Nandi, Jigang Wang, Sascha Preu, Mona Jarrahi

**Affiliations:** 1 Electrical and Computer Engineering Department, University of California, Los Angeles, CA USA; 2 Department of Physics and Astronomy and Ames Laboratory-U.S. DOE, Iowa State University, Ames, IA USA; 3 Department of Electrical Engineering and Information Technology, Technical University Darmstadt, Darmstadt, Germany

**Keywords:** Nanoscale devices, Integrated optics, Optical materials and structures, Nanophotonics and plasmonics, Electronics, photonics and device physics

Correction to: *Nature Communications* 10.1038/s41467-021-24957-1, published online 30 July 2021.

In this article the funding from U.S. Department of Energy was omitted. The original article has been corrected.

