# Supplementary material for: Wavelength conversion through plasmon-coupled surface states
Source: Nat Commun. 2021 Jul 30;12:4641. doi: 10.1038/s41467-021-24957-1 (PMC8324784; doi:10.1038/s41467-021-24957-1)
Supplement: Supplementary file 1 — Supplementary Information [file 41467_2021_24957_MOESM1_ESM.pdf]

Supplementary Information:

Wavelength conversion through plasmon-coupled surface states

Authors: Deniz Turan[1], Ping Keng Lu[1], Nezih T. Yardimci[1], Zhaoyu Liu[2], Liang Luo[2], Joong-Mok Park[2], Uttam Nandi[3], Jigang Wang[2], Sascha Preu[3], and Mona Jarrahi[1,*]

Affiliations:

[1]Electrical and Computer Engineering Department, University of California, Los Angeles, CA, 90095, USA.

[2]Department of Physics and Astronomy and Ames Laboratory-U.S. DOE, Iowa State University, Ames, Iowa 50011, USA.

[3]Department of Electrical Engineering and Information Technology, Technical University Darmstadt, 64283 Darmstadt, Germany.

*Corresponding author. Email: mjarrahi@ucla.edu

Supplementary Note 1: Carrier Lifetime Measurements

Optical pump terahertz probe measurements are performed to measure the carrier lifetime of the undoped InAs and p-type InAs (p = 10^{19} cm^{-3}) layers grown on a semi-insulating GaAs substrate. Both measurements are performed on 1-µm-thick InAs layers. As shown in Supplementary Fig. 7, the carrier lifetime of the p-type InAs layer is measured as 5 ps. The carrier lifetime of the undoped InAs layer is estimated to be much larger than 1 ns.

Supplementary Note 2: Impulse Response Calculation

Impulse response current of the nanoantenna array fabricated on a 100-nm-thick undoped InAs layer grown on a 500-nm-thick p-type InAs (p = 10^{19} cm^{-3}) layer grown on a semi-insulating GaAs substrate is calculated. A three-dimensional optical simulation is first performed using a finite-difference time-domain solver (Lumerical) on one nanoantenna unit cell under a TM-polarized 1550 nm optical excitation and the carrier generation profile, $G(x, y, z)$, is obtained from this simulation. Next, the continuity equation is solved for electron density, $n(x, y, z, t)$, under an impulse optical generation rate $G \cdot \delta(t)$:

$$\frac{\partial n}{\partial t} = \frac{1}{q}\nabla \cdot \mathbf{J}_{\boldsymbol{n}} + G \cdot \delta(t) - \frac{n}{\tau} \quad (1)$$

where $\mathbf{J}_{\boldsymbol{n}}$ is the electron current density in A m^{-2}, $\delta(t)$ is the Dirac-delta function, τ is the electron lifetime in seconds, and q is the electron charge in C. Next, Equation (1) is solved for both undoped InAs and p-type doped InAs regions. Following assumptions are made for electron transport in the undoped InAs region:

1. Electron current is dominated by the drift current in the x-direction.
2. Electrons are assumed to drift at the saturation velocity, i.e., $\mathbf{J}_{\boldsymbol{n}} = q\mathbf{v}_{\boldsymbol{e}}n$, $|\mathbf{v}_{\boldsymbol{e}}| = 10^5$ m s^{-1} [1,2].

3. Since the carrier lifetime of the undoped InAs layer is much larger than the transit time of the photogenerated carriers inside the undoped InAs region that drift to the nanoantenna contact (~1 ps), a carrier lifetime of $\tau \to \infty$ is assumed in the undoped InAs region.

Under these assumptions, Equation (1) in the undoped InAs region is modified to:

$$\frac{\partial n}{\partial t} = |\mathbf{v}_e| \frac{\partial n}{\partial x} + G \cdot \delta(t) \tag{2}$$

Following assumptions are made for electron transport in the p-doped InAs region:

1. Electron current is dominated by the diffusion current in the x-direction, i.e., $J_{n,x} = qD_n \frac{\partial n}{\partial x}$.
2. Diffusion constant of the p-type InAs layer is chosen as $D_n = 1.36 \times 10^{-4}\ \mathrm{m}^2\ \mathrm{s}^{-1}$, based on the experimental results reported in[3]. Brooks-Herring's model is used to account for the additional degradation in mobility due to the high doping density.
3. The carrier lifetime of the p-type InAs layer is chosen as 5 ps, based on the measurement results shown in Supplementary Fig. 7.

Under these assumptions, Equation (1) in the p-type doped InAs region is modified to:

$$\frac{\partial n}{\partial t} = D_n \frac{\partial^2 n}{\partial x^2} + G \cdot \delta(t) - \frac{n}{\tau} \tag{3}$$

Considering current continuity at the interface between the undoped and p-doped InAs layers, Equations (2) and (3) are solved to obtain $n(x, y, z, t)$. The time-evolution of the carrier density, n, is shown in the supplementary movie S2.

Next, the density of the electron current injected to the nanoantenna at each surface point (y, z) is calculated as:

$$J_{\text{injected}}(0, \mathrm{y}, \mathrm{z}, \mathrm{t})\hat{\mathbf{x}} = q|\mathbf{v}_e| n(0, y, z, t)\hat{\mathbf{x}} \tag{4}$$

To calculate the induced current on the nanoantennas, the nanoantenna area is first divided into discrete regions, i, with center locations of $(y_i,\ z_i)$ and dimensions of Δy and Δz. The overall injected current at the ith discrete region is calculated as:

$$\tilde{I}_{\text{injected}}(y_i, z_i, f) = \int_{y_i-\frac{\Delta y}{2}}^{y_i+\frac{\Delta y}{2}} \int_{z_i-\frac{\Delta z}{2}}^{z_i+\frac{\Delta z}{2}} \tilde{J}_{\text{injected}}(0, y, z, f)\,dy\,dz \tag{5}$$

$\tilde{J}_{\text{injected}}$ is the Fourier transform of the calculated time domain current, J_{injected}. The overall induced current on the nanoantennas is computed using a finite-element-method-based electromagnetic solver (ANSYS-HFSS). The calculated injected currents at all of the discrete regions, $\tilde{I}_{\text{injected}}(y_i, z_i, f)$, are included as multiple current sources across the nanoantenna area. One nanoantenna unit-cell is simulated and periodic boundary conditions are used in the y and z directions to account for the impact of the current injected to the adjacent nanoantennas while calculating the induced impulse response current on the nanoantenna, $\tilde{I}_{\text{impulse}}(y, z, f)\hat{\mathbf{z}}$. Supplementary Figs. 8a and 8b show the impulse response current of the nanoantenna array and its Fourier transform amplitude, respectively.

To calculate the overall induced current on the nanoantennas, $\tilde{I}_{\text{induced}}(y,z,f)\hat{\mathbf{z}}$, the obtained impulse response is convolved with the temporal profile of the femtosecond optical pulse. Figs. 4a-f show the overall induced current on the nanoantennas as a function of frequency for different nanoantenna lengths and the decomposition of the overall induced current to the contribution of the injected current from various spots along the antenna length. As the current injection position is moved away from the nanoantenna tip and the nanoantenna-ground line intersection, it splits into two current components in opposite directions and with an approximately equal magnitude. This is because a fraction of the current that is injected to the adjacent nanoantennas flows to the neighboring nanoantennas and induces a current in the opposite direction. To better illustrate this phenomenon, we compare the induced current on a nanoantenna under three different scenarios: (1) when no neighboring nanoantenna is excited with an injected current (Supplementary Fig. 10b); (2) when only the two neighboring nanoantennas are excited with an equal injected current (Supplementary Fig. 10c); and (3) when six neighboring nanoantennas are excited with an equal injected current (Supplementary Fig. 10d). As illustrated in Supplementary Fig. 10b, when no neighboring nanoantenna is excited, the current flow is mostly oriented toward the ground line due to the impedance asymmetry created by the ground line. However, as the adjacent nanoantennas are excited (Supplementary Figs. 10c-d) a portion of their currents flows to the nanoantenna and produces a current flow in the opposite direction with an approximately equal magnitude. Furthermore, there is a rapid drop in the induced current closely after the current injection location. This is because the photogenerated electrons increase the substrate conductivity, which results in a fraction of the injected current leaking to the substrate, as illustrated in Supplementary Fig 10a.

Supplementary Note 3: Radiation Power Calculation

To calculate the radiated power from the nanoantenna array, the radiation field of a single nanoantenna, $\mathbf{E_i}(r,\theta,\phi,f)$, is calculated first. Since the nanoantenna length is much smaller than the radiation wavelength and since most of the radiated power flows toward the semiconductor substrate, the vector potential can be written as[4]:

$$A_z(r,f) = \frac{\mu_0}{4\pi}\frac{e^{-jkr}}{r}\int_0^{L_a}\int_0^{W_a}\tilde{J}_{\text{induced}}(y',z',f)dy'dz' = \frac{\mu_0}{4\pi}\frac{e^{-jkr}}{r}S(L_a,W_a) \qquad (6)$$

where $\tilde{J}_{\text{induced}}(y,z,f)$ is the induced surface current on the nanoantennas with an Am^{-1} unit, L_a and W_a are the length and width of each nanoantenna, respectively, $S(L_a,W_a)$ is the result of the integral, μ_0 is the permeability of the free space, $k = 2\pi f/c$ is the free space wavenumber, $r = \sqrt{x^2+y^2+z^2}$ is the observation point distance.

From the vector potential expression, the far-field radiated electric field for one nanoantenna can be written as[4]:

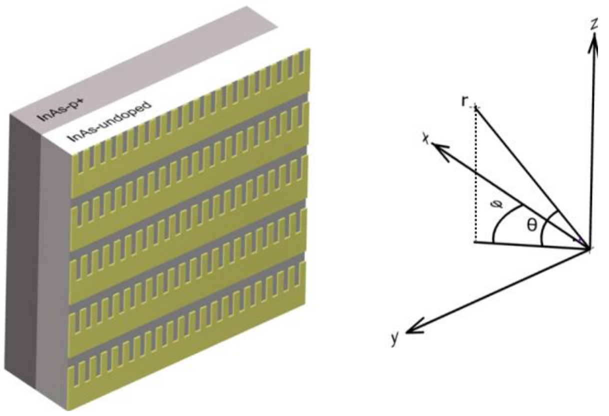

$$\mathbf{E_i}(r,\theta,\phi,f) \approx j\eta_0 k\sin\theta \frac{e^{-jkr}}{4\pi r} S(L_a, W_a)\hat{\boldsymbol{\theta}} \tag{7}$$

where η_0 is the wave impedance of the free space. Next, the radiation pattern of the nanoantenna array is calculated using the radiation pattern of one nanoantenna, $\mathbf{E_i}(r,\theta,\phi,f)$ [4]:

$$\mathbf{E_{array}}(r,\theta,\phi,f) = \mathbf{E_i}(r,\theta,\phi,f) AF_y(\theta,\phi,f) AF_z(\theta,f) \tag{8}$$

where AF_y and AF_z are the array factor of the nanoantenna array in the y and z directions:

$$AF_y(\theta,\phi,f) = \frac{\sin\left(\frac{N_y\psi_y}{2}\right)}{\sin\left(\frac{\psi_y}{2}\right)}, \psi_y = p_y k\sin\phi\sin\theta \tag{9}$$

$$AF_z(\theta,f) = \frac{\sin\left(\frac{N_z\psi_z}{2}\right)}{\sin\left(\frac{\psi_z}{2}\right)}, \psi_z = p_z k\cos\theta \tag{10}$$

where p_y and p_z are the periodicity of the nanoantennas in the y and z directions, respectively, N_y and N_z are the number of nanoantennas in the y and z directions, respectively, and $k = 2\pi n f/c$ is the wavenumber at a given radiation wavelength ($n = n_{GaAs} = 3.6$). Finally, the radiated power from the nanoantenna array that propagates toward the semiconductor substrate is calculated as:

$$P(f) = \frac{1}{2\eta_0} \int_{-\pi/2}^{\pi/2} \int_0^{\pi} \left|\mathbf{E_{array}}(\theta,\phi,f)\right|^2 r^2 \sin\theta d\theta d\phi \tag{11}$$

Supplementary Note 4: Surface Plasmon Excitation
Periodicity of the nanoantennas in the y-direction is chosen as 440 nm to provide the necessary momentum to couple the photo-excited surface plasmon waves to the interface between the nanoantennas and the InAs substrate when excited by a TM-polarized optical beam at a 1550 nm wavelength (Supplementary Fig. 6a). A 240-nm-thick Si_3N_4 anti-reflection coating, a 360-nm-thick nanoantenna width, and a 3/97-nm-thick Ti/Au nanoantenna height are used to increase the coupling efficiency of surface plasmon waves. To illustrate the impact of the excited surface plasmon waves, an alternative nanoantenna geometry is analyzed, which has a periodicity of 160 nm in the y-direction. The momentum provided by this periodicity is larger than the momentum required for the excitation of surface plasmon waves (Supplementary Fig. 6b). Although the optical transmission to the InAs layer provided by this nanoantenna (Supplementary Fig. 6c) is much higher than that of the plasmonic nanoantenna, the plasmonic nanoantenna provides 7 times higher optical absorption within a 100 nm depth inside the InAs layer, where the built-in electric field strength is maximized (Supplementary Fig. 6d).

To quantify the overlap between the optical absorption and built-in electric field, an overlap integral function, $OI(x)$, is defined as:

$$OI(x) = \frac{\int_0^x \int_0^{w_a} E_{\mathrm{bi}}(y', x') I_{\mathrm{abs}}(x', y') dy' dx'}{\int_0^{\Lambda} I_{\mathrm{in}}(y') dy'} \quad (12)$$

where E_{bi} is the built-in electric field shown in Fig. 3c color plots, I_{abs} is the absorbed optical beam intensity at a 1550 nm wavelength (Supplementary Figs. 6a and 6b insets), I_{in} is the incident optical beam intensity on the nanoantenna array, and x is the depth in the InAs substrate. As expected, the calculated overlap integral values shown in Supplementary Fig. 6e, are consistently higher for the nanoantenna design that supports surface plasmon waves.

The coupling efficiency of surface plasmon waves can be further enhanced by increasing the height of the nanoantennas. Supplementary Fig. 14a shows how the optical absorption within a 100 nm depth in InAs changes as a function of the nanoantenna height for the same nanoantenna periodicity. While nanoantenna heights larger than 100 nm can provide higher photoabsorption peaks[5], the redshift in the absorption peak prevents any increase in the photoabsorption in the laser operation wavelength range of 1550-1560 nm. This redshift is caused by the relatively longer propagation path length of the excited surface waves on the plasmonic nanoantennas when the metal height is increased, which results in a shift in the dispersion curve of the nanoantenna arrays[6]. The shift in the dispersion curve can be compensated by adjusting the nanoantenna periodicity, Λ, to keep the absorption peak in the laser operation wavelength range. As shown in Supplementary Fig. 14b, a nanoantenna periodicity of 420 nm and nanoantenna height of 140 nm can offer 39% optical absorption within a 100 nm depth in InAs, which is 44% higher than the absorption provided by the nanoantenna used in our demonstration with a periodicity of 440 nm and metal height of 100 nm. The 44% increase in optical absorption would increase the wavelength conversion efficiency by 100% at low optical powers (quadratic regime) and 44% at high optical powers (linear regime).

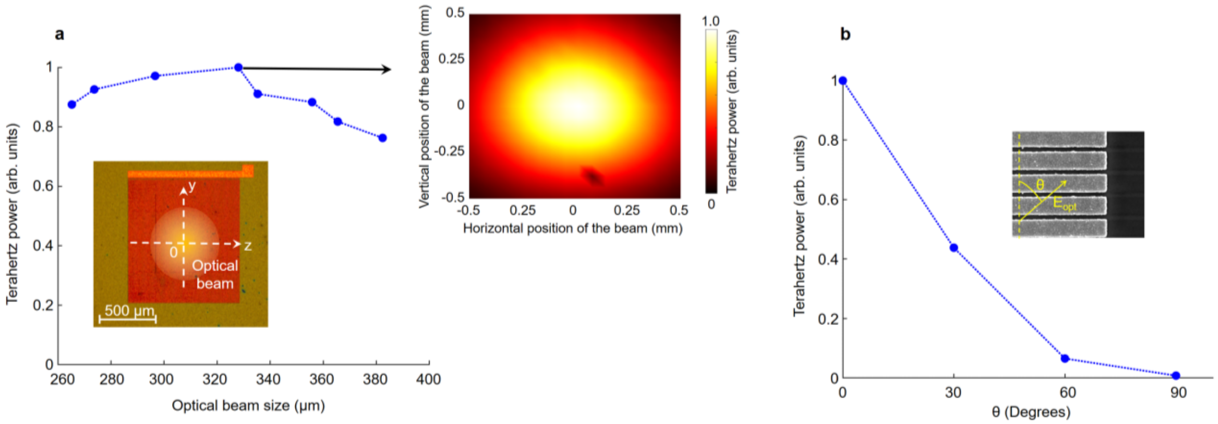

Supplementary Fig. 1. Optical beam size, position, and polarization requirements. (a) The measured terahertz radiation power from the nanoantenna array for different optical beam sizes and beam positions. Beam size is defined as the beam diameter at which the optical intensity drops to $1/e^2$ (13.5%) of its peak value. The measurement results show less than a 25% reduction in the radiation power when the optical beam size is deviated from the optimum value (330 μm) by 50 μm. When the optical beam size is reduced relative to this optimum value, the wavelength conversion efficiency is reduced due to the carrier screening effect. When the optical beam size is increased relative to this optimum value, the wavelength conversion efficiency is reduced due to the destructive interference of the radiation from the relatively distant nanoantennas. The results also show less than a 25% reduction in the radiation power when the optical beam position is deviated from the optimum position (center of the nanoantenna array) by 250 μm. Therefore, wavelength conversion through plasmon-coupled surface states has a high tolerance for optical beam size variations and misalignment. **(b)** The measured terahertz radiation power from the nanoantenna array as a function of optical polarization. The wavelength conversion efficiency drops when deviating from the optimum polarization for the excitation of surface plasmons (orthogonal to the nanoantennas) due to the reduction in the number of the photogenerated electrons at the semiconductor surface.

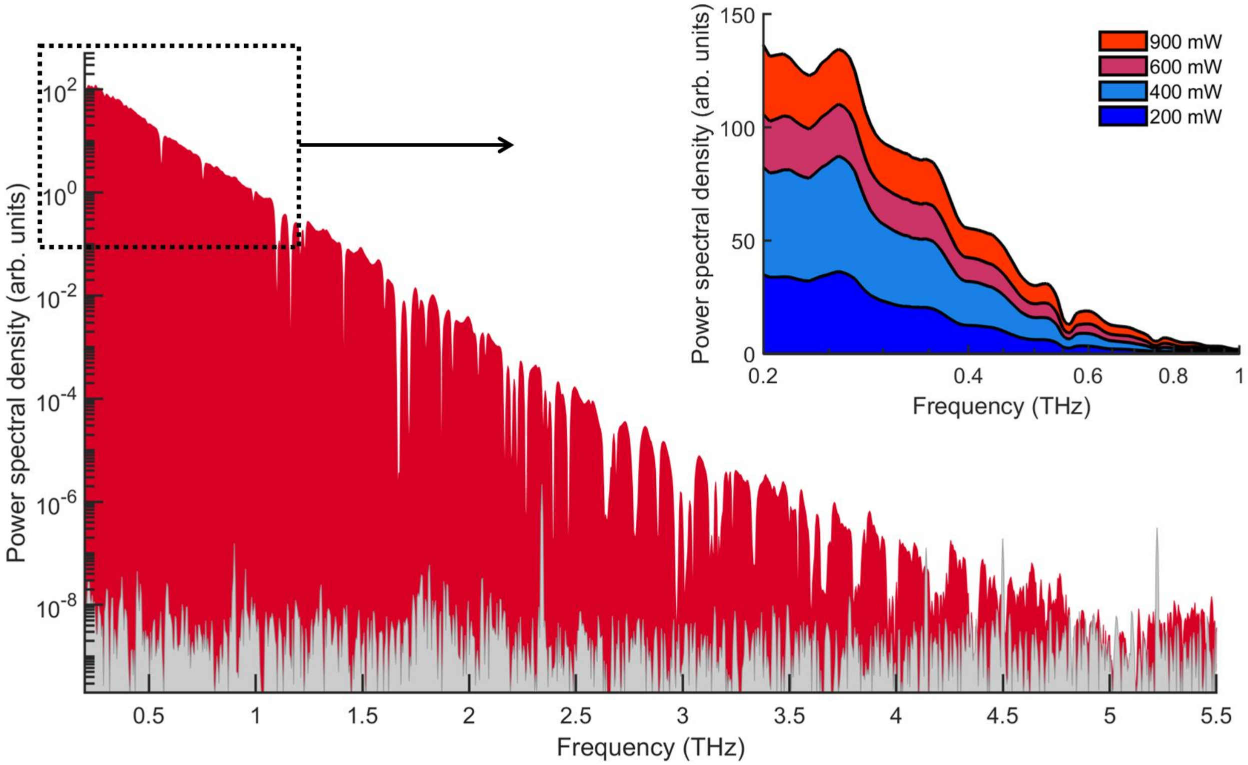

Supplementary Fig. 2. Terahertz radiation spectrum. The measured terahertz radiation spectrum (shown in red) along with the noise spectrum (shown in gray) when the nanoantenna array is excited by optical pulses with a 1550 nm center wavelength, 900 mW power, 120 fs pulsewidth, and 76 MHz repetition rate. 650 time-domain traces are captured and averaged to resolve this terahertz spectrum. Dependence of the radiation spectrum on the optical power level is shown in the inset. The impact of the optical power on the generated terahertz spectrum is investigated for the nanoantenna array with a 2 μm nanoantenna length, 2 μm ground line width, and 0.5 μm gap between the nanoantenna array rows, fabricated on a 100-nm-thick undoped InAs layer grown on a 500-nm-thick p-type (10^{19} cm^{-3}) InAs epilayer on a semi-insulating GaAs substrate. As shown in Fig. 2d and Supplementary Fig. 4, the generated terahertz power level has a quadratic dependence on the optical power level at low optical power levels (< 10 mW), a linear dependence at higher optical power levels (>50 mW), and saturation behavior at 900 mW optical power. This change in power dependence is due to the high peak power of the femtosecond optical pulses, which results in an excessive number of free carriers in the InAs layer, screening the built-in electric field.

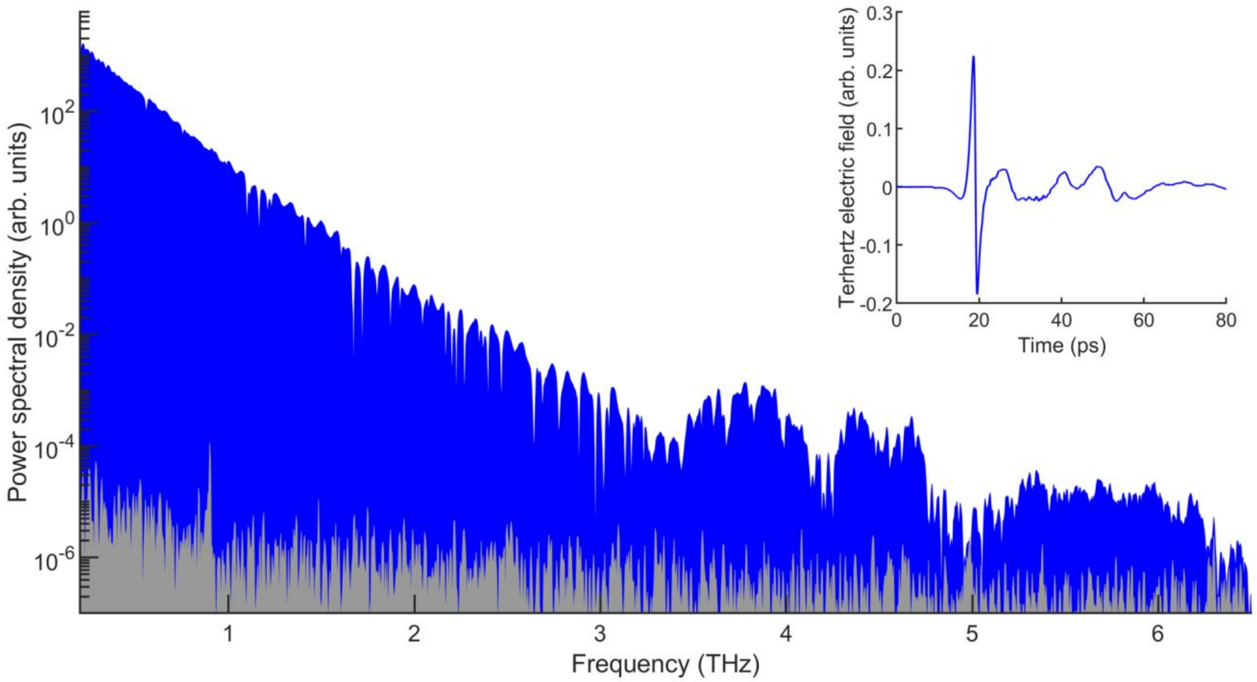

Supplementary Fig. 3. Terahertz radiation spectrum under 23 fs optical pulsewidth. The measured terahertz radiation spectrum (shown in blue) along with the noise spectrum (shown in gray) when the nanoantenna array is excited by optical pulses with a 1560 nm center wavelength, 100 mW power, and 23 fs pulsewidth. 1000 time-domain traces are captured and averaged to resolve this terahertz spectrum. The time-domain radiated terahertz pulse is shown in the inset.

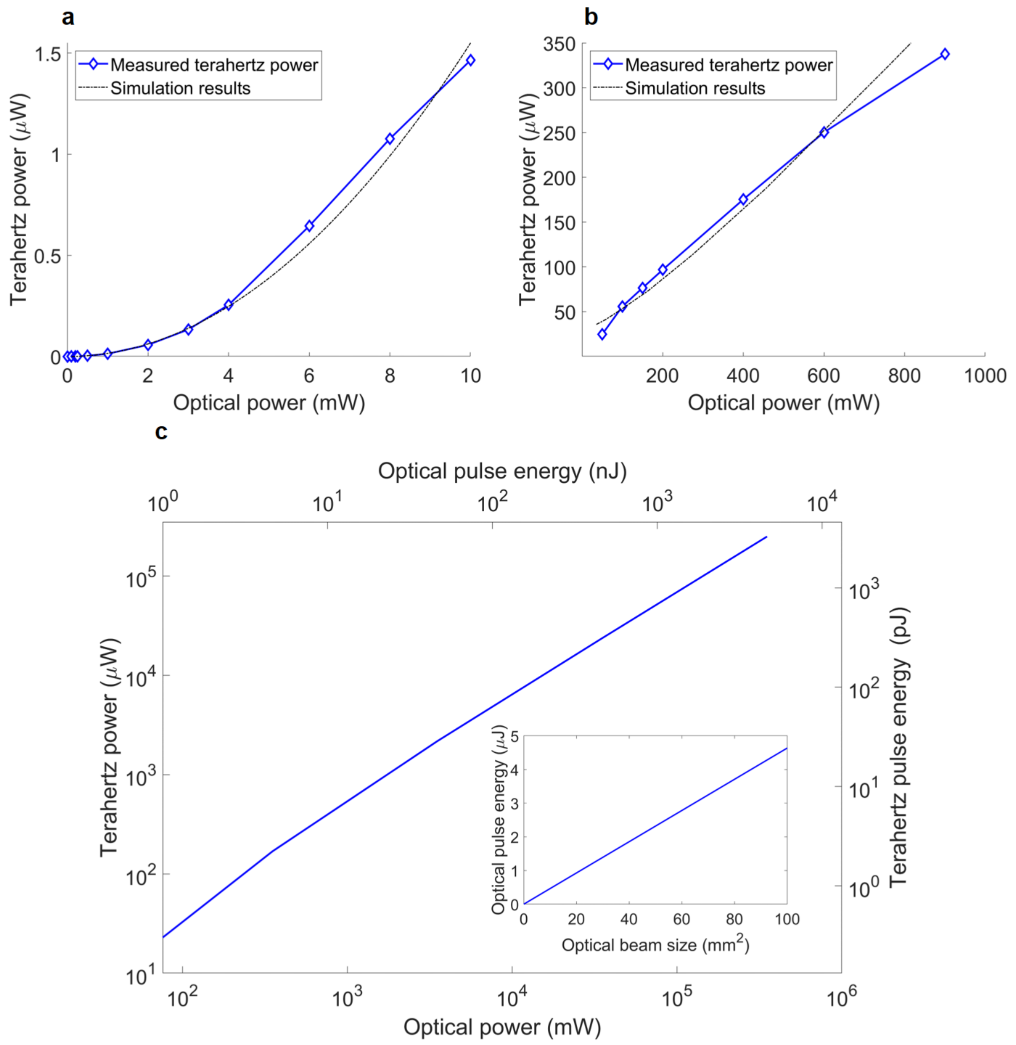

Supplementary Fig. 4. Dependence of the converted power on the optical power under pulsed excitation. (a) For optical powers below 10 mW where a quadratic dependence is observed and **(b)** optical powers above 50 mW where a linear dependence is observed and saturation starts impacting the power at 900 mW. Dashed lines show the calculated converted power. The physical factor that determines the power dependence of the presented wavelength conversion scheme (quadratic, linear, or saturation) is the carrier screening effect. When the photo-generated carriers inside the semiconductor active area drift to the nanoantennas by the built-in electric field, the separated electron-hole pairs create an imbalance in charge density. This imbalance induces an electric field in the opposite direction relative to the built-in electric field. This screening field is proportional to the photo-generated carrier density in the substrate, as predicted by the Gauss's Law. Since the generated carrier density is proportional to the optical intensity incident on the nanoantenna array, the carrier screening effect is negligible at low optical power levels (screening field << built-in field). In this regime, the generated carrier density and induced current on the nanoantennas, hence, the radiated field have a linear dependence on the optical power level. Therefore, the radiated power from the nanoantenna array, which has a quadratic dependence on the radiated field has a quadratic dependence on the optical power level in the low optical power regime as described by Eq. 11. As the screening field becomes comparable with the built-in electric

field at higher optical powers, the carrier screening effect starts to limit the increase of the injected current from the substrate to the nanoantennas and the radiated power from the nanoantennas, changing the radiated power dependence on the optical power level from quadratic to linear and then saturation. To reduce the impact of the carrier screening effect on the wavelength conversion efficiency at high optical powers, the spot size of the optical beam incident on the nanoantenna arrays is increased so that the optical intensity is kept below the saturation regime at each power level. This beam size adjustment varies the number of the radiating nanoantennas while maintaining the same induced current on each nanoantenna and, thus, the same radiated field by each nanoantenna, $\mathbf{E_i}(r, \theta, \phi, f)$. To predict the generated terahertz power in this regime the radiated field by the nanoantenna array is computed by Eqs. 8-10. By adjusting the beam size, the radiating nanoantenna array size in the y and z directions (N_y and N_z) is changed in Eqs. 9 and 10. Finally, the radiated power at different terahertz frequencies is computed by calculating $P(f)$ using Eq. 11. **(c)** The calculated total radiated power, $\int P(f)df$, indicating a linear dependence on the optical power level beyond 50 mW, when the optical beam size incident on the nanoantennas is linearly increased as a function of the optical power level (inset). While the maximum optical power and pulse energy offered by our laser (1 W and 10 nJ) do not cover the entire simulated range, the simulation results match the experimental results within our laser operation range (Fig. 2d). A linear increase in the optical spot size as a function of the optical power maintains the optical intensity incident on the nanoantennas below the saturation regime and provides a linear relation between the radiated power and optical power at high power levels. In other words, the nanoantenna array behaves as an effective radiating aperture, which maintains the same radiated field intensity in the aperture while having an aperture area that scales linearly as a function of the optical power level, resulting in a linear relation between the total radiation power and the optical power level. Therefore, there is no limitation in the operating power range of the presented wavelength conversion scheme: as far as the nanoantenna array area can be increased linearly with the optical power level, the radiated power is increased linearly with the optical power level. While nanoantenna array areas of a few mm^2 can be easily patterned using electron-beam lithography, nanoimprint lithography and laser interference lithography are better alternatives for patterning larger size nanoantenna arrays.

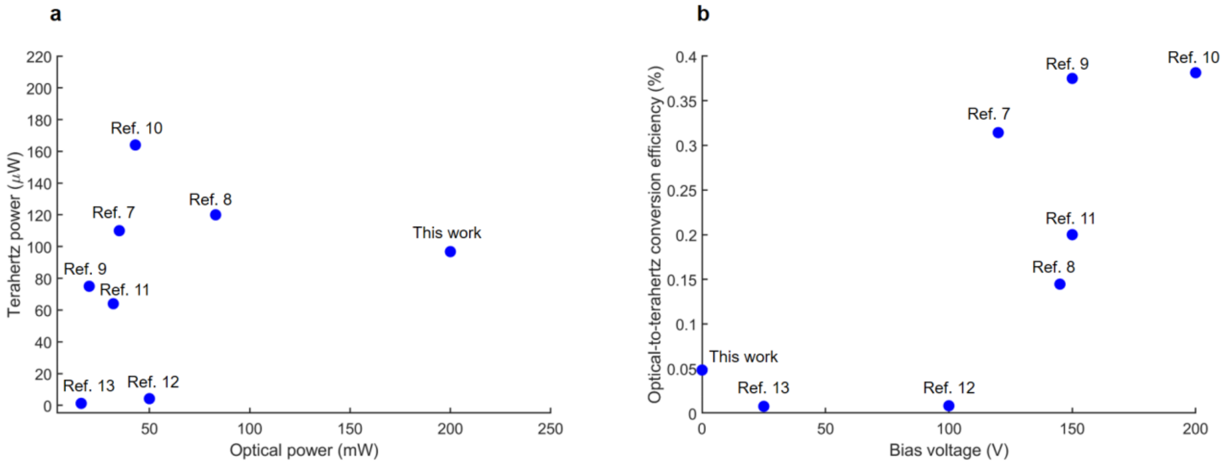

Supplementary Fig. 5. Performance comparison with active optical-to-terahertz converters. Comparison between the performance of the fabricated passive nanoantenna array with the reported active optical-to-terahertz converters operating at 1550 nm wavelength [7–13]. Considering the passive nature of the nanoantenna array, a comparison with other passive optical-to-terahertz converters provides a better assessment, which is shown in Fig. 2d. However, the comparison with active optical-to-terahertz converters is shown here to point out the electrical requirements of the active optical-to-terahertz converters to radiate similar terahertz power levels. **(a)** The radiated terahertz power as a function of optical power, **(b)** The optical-to-terahertz conversion efficiency as a function of the bias voltage. While the power efficiency of the passive nanoantenna array (bias voltage = 0, electrical power consumption = 0) is the same as its optical-to-terahertz conversion efficiency, the power efficiency of the active optical-to-terahertz converters (bias voltage ≠ 0, electrical power consumption ≠ 0) are much lower than their optical-to-terahertz conversion efficiency since considerable electrical current (mA-level) and bias voltage (100-200 V) are required to electrically pump these devices. The large electrical current and voltage levels required for pumping active optical-to-terahertz converters also degrade their reliability.

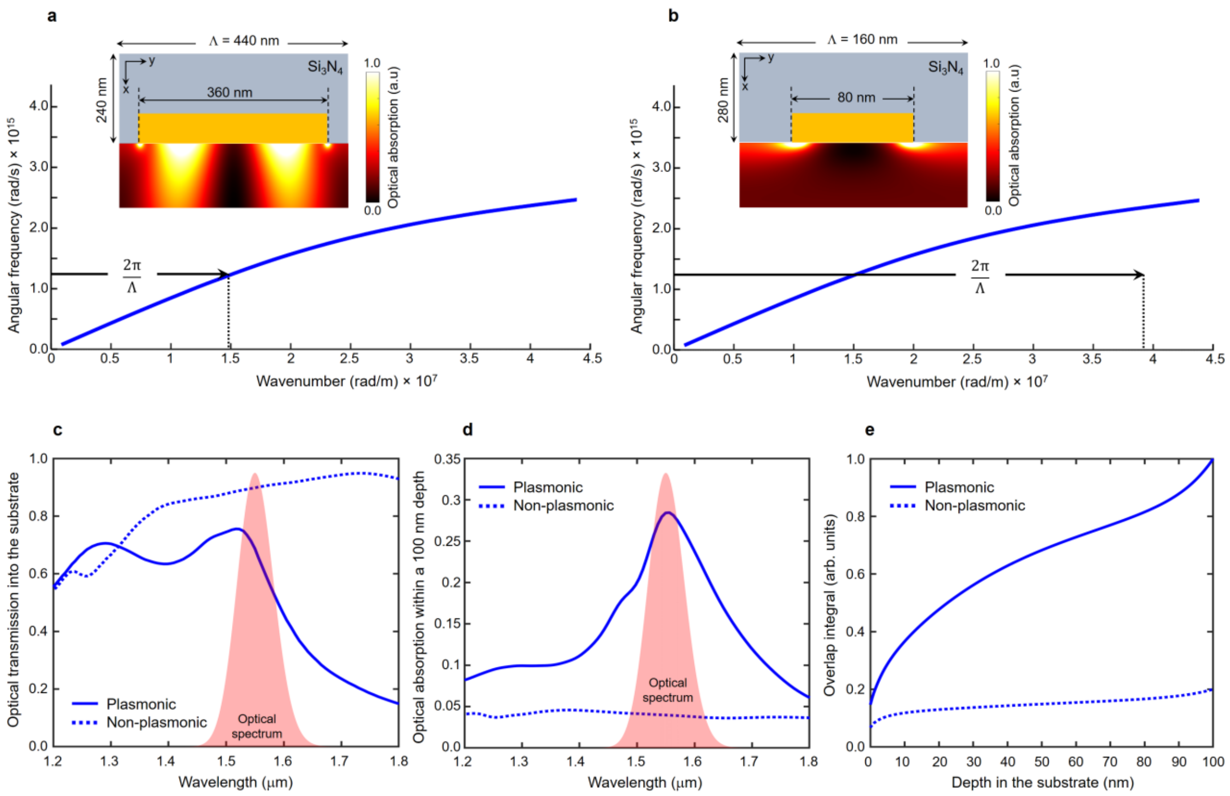

Supplementary Fig. 6. Surface plasmon excitation. (a) Surface plasmon dispersion curve and the momentum provided by nanoantennas with a periodicity of Λ = 440 nm. Optical absorption profile inside the InAs layer when a 1550 nm TM-polarized optical excitation is incident on the nanoantennas (inset). **(b)** Surface plasmon dispersion curve and the momentum provided by nanoantennas with a periodicity of Λ = 160 nm. Optical absorption profile inside the InAs layer when a 1550 nm TM-polarized optical excitation is incident on the nanoantennas (inset). Optical transmission spectrum to the InAs layer, optical absorption spectrum within a 100 nm depth in the InAs layer, and the overlap integral calculated as a function of depth in the substrate for both nanoantenna designs are shown in **(c)**, **(d)**, and **(e)**, respectively. All of the optical simulations are performed using a finite-difference time-domain solver (Lumerical).

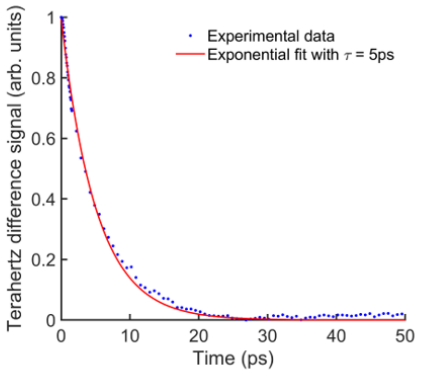

Supplementary Fig. 7. Optical pump terahertz probe measurement of the semiconductor substrate. The measurements are performed on the p-type InAs (p = 10^{19} cm^{-3}) layer grown on a semi-insulating GaAs substrate.

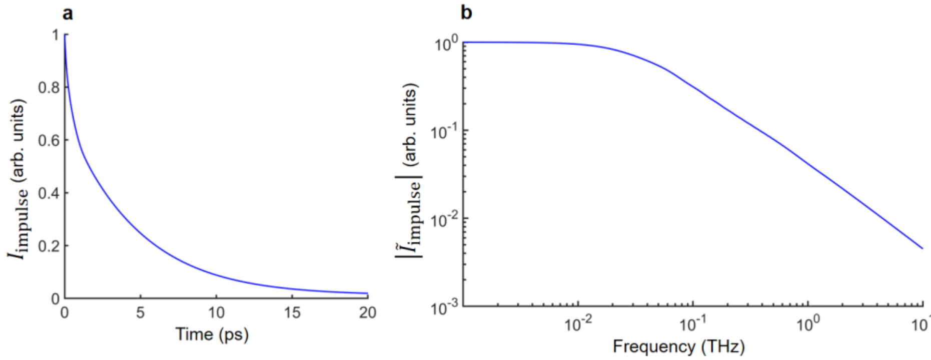

Supplementary Fig. 8. Impulse response of the nanoantenna array. Impulse response current of the nanoantenna array and its Fourier transform amplitude are shown in **(a)** and **(b)**, respectively.

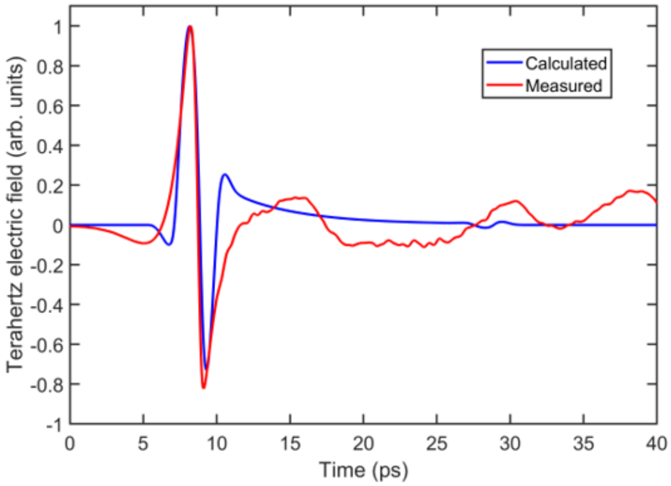

Supplementary Fig. 9. Time-domain terahertz field. Comparison between the calculated (blue) and measured (red) radiated field. The measured full-width at half maximum (FWHM) pulse widths are 1.1 ps and 1.0 ps in the positive and negative cycles. The estimated radiated pulse widths are calculated by convolving the impulse response current of the nanoantenna array (shown in Supplementary Fig. 8) with the temporal profile of the femtosecond optical pulse to calculate the overall induced current on the nanoantennas. The frequency components of the induced current on the nanoantennas are obtained by taking the Fourier transform of the time-domain current. Next, the frequency-domain nanoantenna current is multiplied by the frequency response of the radiated field from the nanoantenna array. Finally, the time-domain radiated field is calculated by taking the inverse Fourier transform of the frequency-domain results. The calculated FWHM pulse widths of the positive and negative pulses are 1.1 ps and 0.8 ps, respectively, showing a close agreement between the calculated and measured data. The small broadening in the measured negative pulse and slow following oscillations are due to the low frequency oscillations induced in the output circuit of the photoconductive detector used for measuring the radiated field.

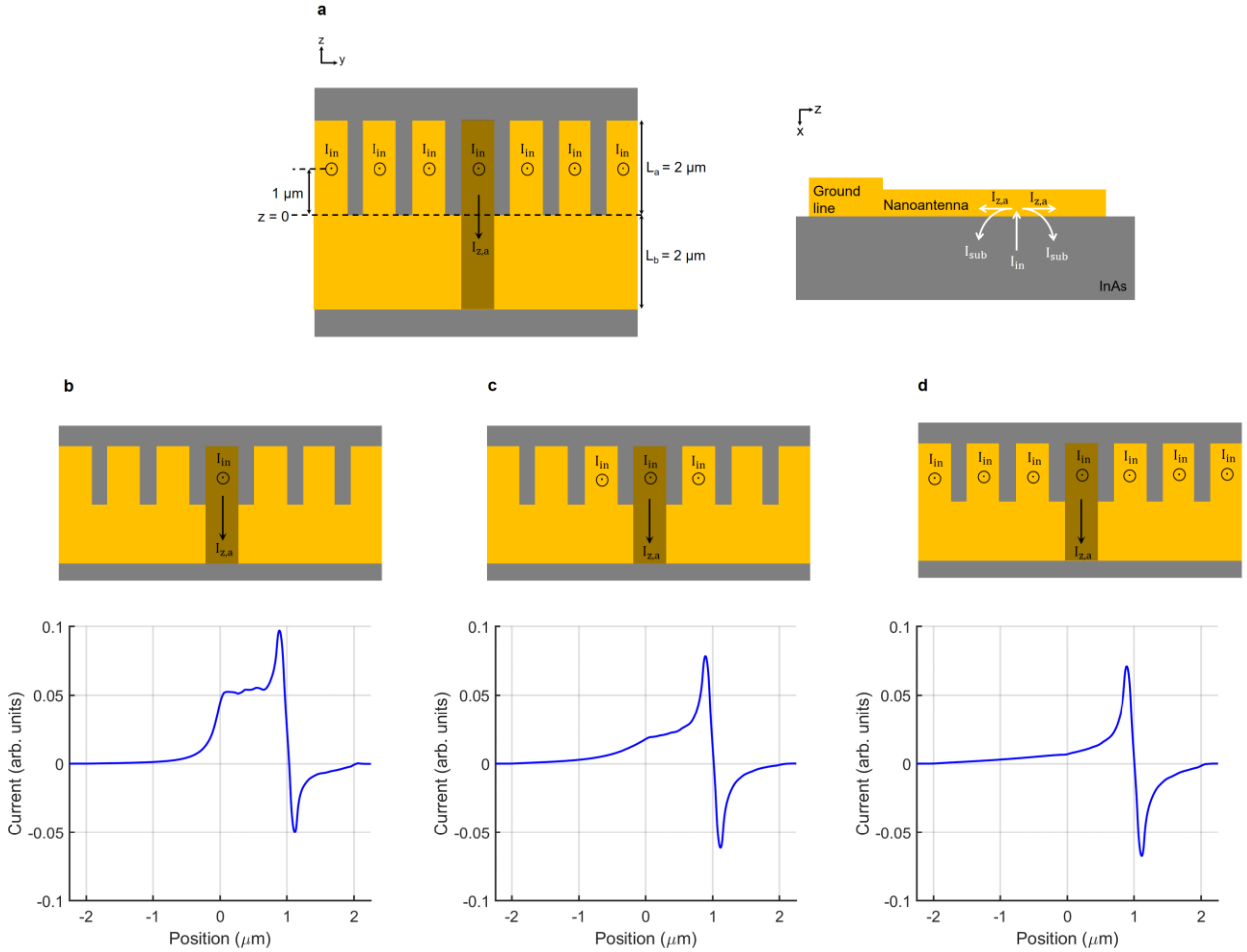

Supplementary Fig. 10. Coupling between the nanoantenna array elements. (a) Top view and side view of the simulated nanoantenna array to investigate the induced current on each nanoantenna. The induced current on the shaded nanoantenna when **(b)** the neighboring nanoantennas are not excited, **(c)** the two neighboring nanoantennas are excited, and **(d)** six neighboring nanoantennas are excited.

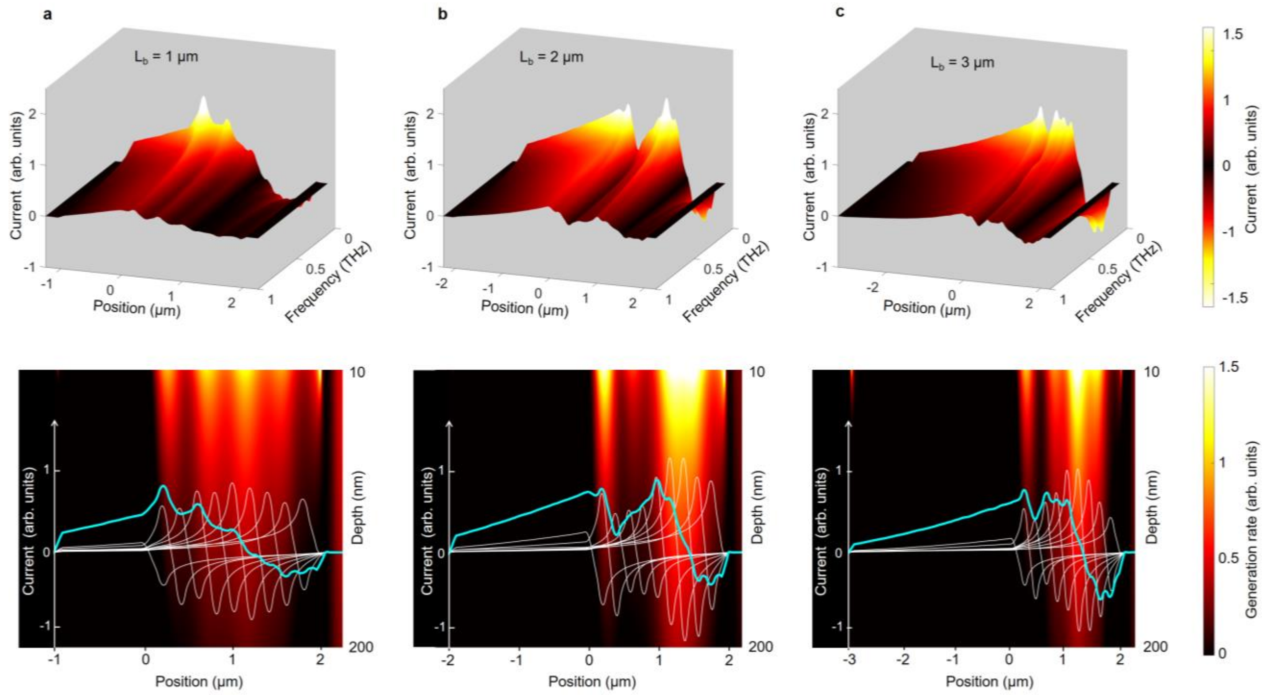

Supplementary Fig. 11. Impact of the nanoantenna ground line width on the wavelength conversion efficiency. (a-c) Top: The induced current on the nanoantennas as a function of frequency when the ground line width (L_b) is varied from 1 μm to 3 μm. The nanoantenna length (L_a) and the gap between the nanoantenna array rows (L_g) are chosen as 2 μm and 0.5 μm, respectively. The ground line junction with the nanoantenna is located at the 0 position and the nanoantenna is located between 0 and 2 μm positions along the z-axis. a-c Bottom: Decomposition of the total induced current on the nanoantennas (teal lines) to the individual contributions of the injected currents from different positions of the nanoantennas (white lines) at 0.2 THz. The background color maps show the electron generation profiles averaged over the nanoantenna width.

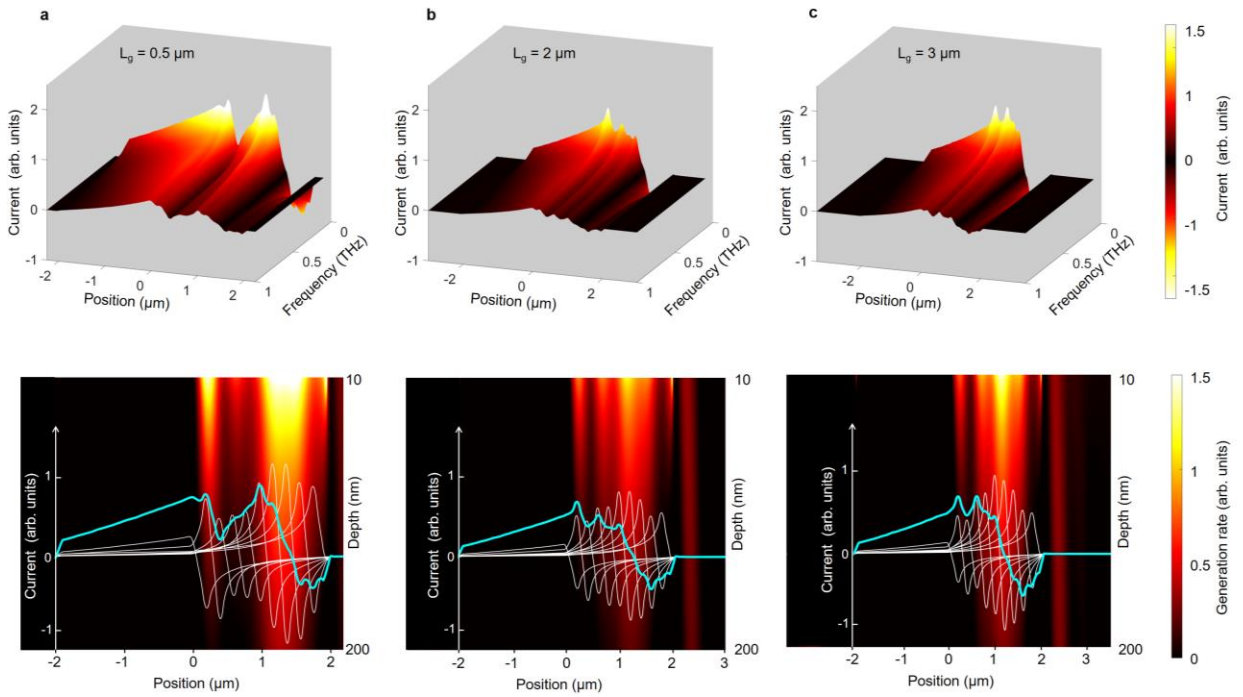

Supplementary Fig. 12. Impact of the gap between the nanoantenna array rows on the wavelength conversion efficiency. (a-c) Top: The induced current on the nanoantennas as a function of frequency when the gap between the nanoantenna array rows (L_g) is varied from 0.5 μm to 3 μm. The nanoantenna length (L_a) and the ground line width (L_b) are chosen as 2 μm and 2 μm, respectively. The ground line is located between -2 μm and 0 positions and the nanoantenna is located between 0 and 2 μm positions along the z-axis. a-c Bottom: Decomposition of the total induced current on the nanoantennas (teal lines) to the individual contributions of the injected currents from different positions of the nanoantennas (white lines) at 0.2 THz. The background color maps show the electron generation profiles averaged over the nanoantenna width.

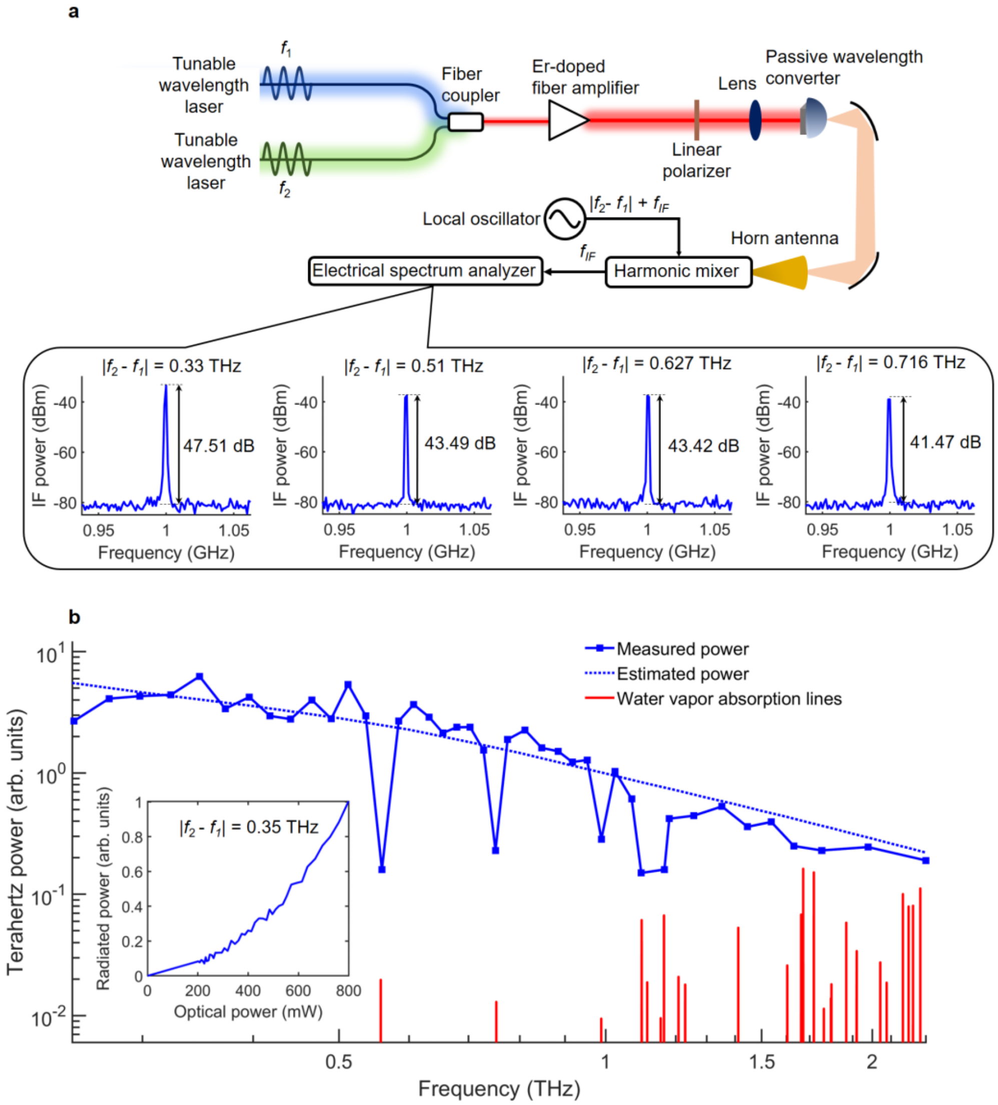

Supplementary Fig. 13. Continuous-wave optical-to-terahertz conversion. (a) Top: Experimental setup used to measure CW radiation from the nanoantenna array. The nanoantenna array is excited by two 1550 nm CW lasers (Santec TSL-510 and New Focus TLB-6730-P) with equal optical power levels. The radiation frequency is tuned by adjusting the beat frequency of the two lasers. In this experimental setup, the beat frequency is varied between 0.23 THz and 2.3 THz. The generated radiation from the nanoantenna array is routed to a harmonic mixer (VDI MixAMC with WR 1.5 and WR 2.2 waveguides) to down-convert it to an intermediate frequency (IF) signal in the GHz frequency range. The IF signal for each optical beat frequency is measured using an electrical spectrum analyzer (HP8592L), as shown in the inset. **(b)** The measured radiation power from the nanoantenna array over the 0.23-2.3 THz frequency range using a pyroelectric detector. The observed power roll-off as a function of frequency agrees with the roll-off predicted by the calculated impulse response. The deviations at low frequencies are due to the poor coupling of the radiation to the pyroelectric detector. The dips in the spectrum match the water vapor absorption lines[14], shown in red. The inset shows the dependence of the radiated power on the incident optical power at 0.35 THz. Unlike the pulsed operation mode, the radiated power follows a quadratic relation with the incident optical power.

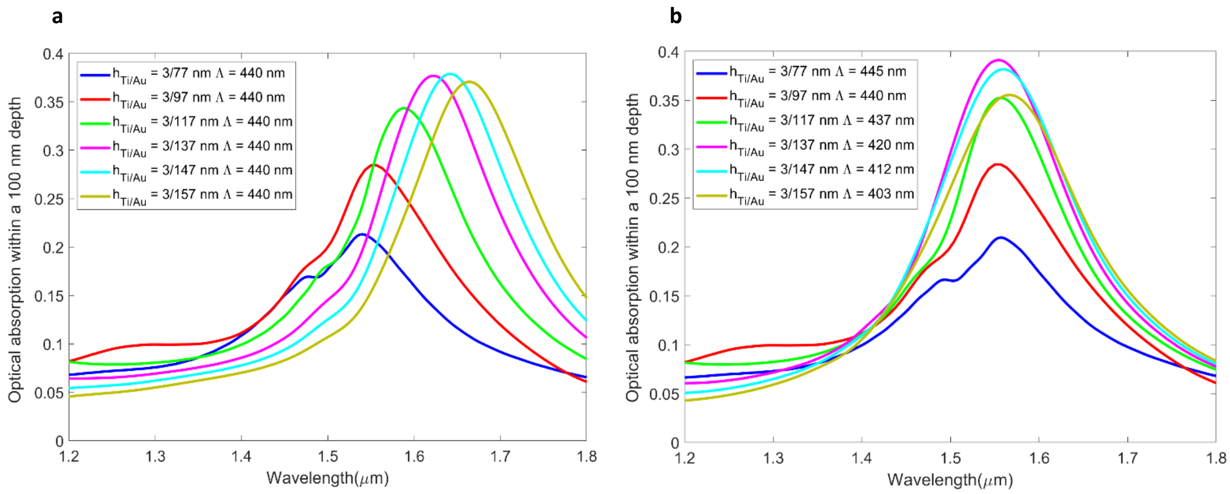

Supplementary Fig. 14. Impact of metal thickness on the wavelength conversion efficiency. Optical absorption within a 100 nm depth in InAs, where the built-in electric field intensity is maximized for **(a)** nanoantenna arrays with a periodicity of Λ = 440 nm and Ti/Au metal thicknesses varying from 3/77 nm to 3/157 nm, **(b)** nanoantenna arrays with periodicities ranging from 445 nm to 403 nm and Ti/Au metal thicknesses varying from 3/77 nm to 3/157 nm. The nanoantenna periodicity is adjusted to keep the absorption peak in the laser operation wavelength range of 1550-1560 nm.

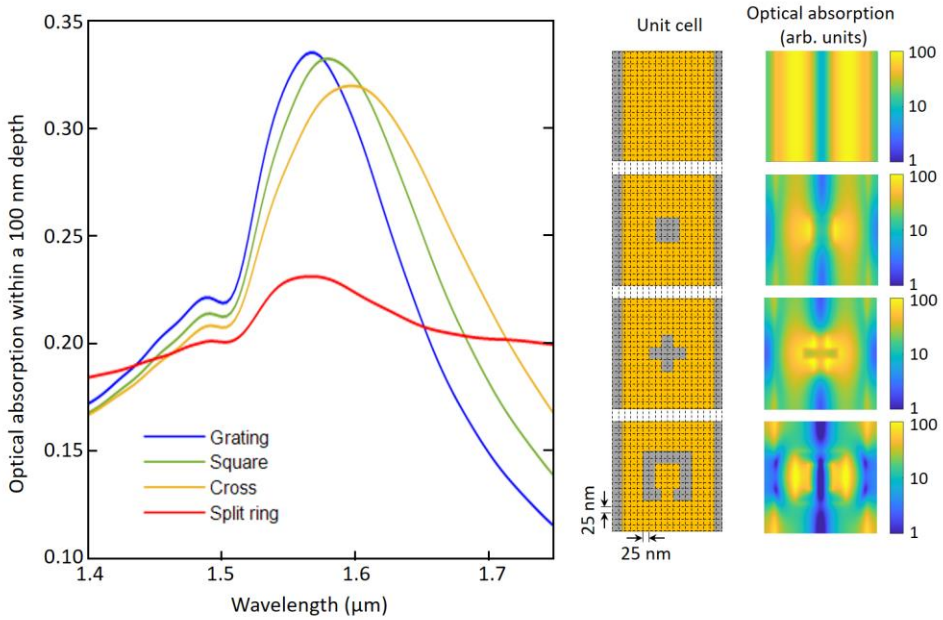

Supplementary Fig. 15. Plasmonic nanoantenna design flexibility. Optical absorption within a 100 nm depth in InAs where the built-in electric field intensity is maximized for grating, split-ring, square, and cross-shaped structures plasmonic nanoantenna architectures (calculated by Lumerical). A 100 nm-thick metal is used in all of the simulations. The geometry of the simulated structures and the optical absorption profile in InAs for each structure are shown on the right. The results show that different nanoantenna structures can be used for coupling surface plasmons to the surface states, where the built-in electric field is maximized. While the square and cross-shaped geometries offer similar optical absorption compared to the grating-shaped structure, the grating-shaped nanoantennas (the structure used in the fabricated device) offer the highest optical absorption, which directly translates to the highest wavelength conversion efficiency.

Supplementary References:

1. Satyanadh, G., Joshi, R. P., Abedin, N. & Singh, U. Monte Carlo calculation of electron drift characteristics and avalanche noise in bulk InAs. *J. Appl. Phys.* **91**, 1331–1338 (2002).
2. Brennan, K. & Hess, K. High field transport in GaAs, InP and InAs. *Solid State Electron.* **27**, 347–357 (1984).
3. Kalem, S., Chyi, J. I., Morkoç, H., Bean, R. & Zanio, K. Growth and transport properties of InAs epilayers on GaAs. *Appl. Phys. Lett.* **53**, 1647–1649 (1988).
4. Balanis, C. *Antenna Theory; Analysis and Design*. (John Wiley & Sons Inc, Hoboken, NJ, 2005).
5. Hsieh, B. Y. & Jarrahi, M. Analysis of periodic metallic nano-slits for efficient interaction of terahertz and optical waves at nano-scale dimensions. *J. Appl. Phys.* **109**, (2011).
6. Porto, J. A., García-Vidal, F. J. & Pendry, J. B. Transmission resonances on metallic gratings with very narrow slits. *Phys. Rev. Lett.* **83**, 2845–2848 (1999).
7. Globisch, B. *et al.* Absolute terahertz power measurement of a time-domain spectroscopy system. *Opt. Lett.* **40**, 3544–3547 (2015).
8. Mingardi, A. *et al.* High power generation of THz from 1550-nm photoconductive emitters. *Opt. Express* **26**, 14472–14478 (2018).
9. Globisch, B. *et al.* Iron doped InGaAs: Competitive THz emitters and detectors fabricated from the same photoconductor. *J. Appl. Phys.* **121**, 053102 (2017).
10. Nandi, U. *et al.* ErAs:In(Al)GaAs photoconductor-based time domain system with 4.5 THz single shot bandwidth and emitted terahertz power of 164 µW. *Opt. Lett.* **45**, 2812–2815 (2020).
11. Dietz, R. J. B. *et al.* 64 µW pulsed terahertz emission from growth optimized InGaAs/InAlAs heterostructures with separated photoconductive and trapping regions. *Appl. Phys. Lett.* **103**, 061103 (2013).
12. Wood, C. D. *et al.* Terahertz emission from metal-organic chemical vapor deposition grown Fe:InGaAs using 830 nm to 1.55 µm excitation. *Appl. Phys. Lett.* **96**, 194104 (2010).
13. Roehle, H. *et al.* Next generation 1.5 µm terahertz antennas: mesa-structuring of InGaAs/InAlAs photoconductive layers. *Opt. Express* **18**, 2296–2301 (2010).
14. Gordon, I. E. *et al.* The HITRAN2016 molecular spectroscopic database. *J. Quant. Spectrosc. Radiat. Transf.* **203**, 3–69 (2017).
