# Supplementary material for: Wavelength conversion through plasmon-coupled surface states
Source: Nat Commun. 2021 Jul 30;12:4641. doi: 10.1038/s41467-021-24957-1 (PMC8324784; doi:10.1038/s41467-021-24957-1)
Supplement: Supplementary file 2 — Description of Additional Supplementary Files [file 41467_2021_24957_MOESM2_ESM.pdf]

Description of Additional Supplementary Files

File name: Supplementary Movie 1
Description: Illustration of free carrier generation and transport dynamics inside the InAs lattice, where a nanoantenna array couples photo-excited surface plasmons to the surface states.

File name: Supplementary Movie 2
Description: Time-evolution of the photo-generated carrier density inside the InAs layer.
